# An international observational study validating gene-expression sepsis immune subgroups

**DOI:** 10.1186/s13054-025-05319-5

**Published:** 2025-03-03

**Authors:** David B. Antcliffe, Estelle Peronnet, Frédéric Pène, Kristoffer Strålin, David Brealey, Sophie Blein, Richard Cleaver, Maria Cronhjort, Jean-Luc Diehl, Guillaume Voiriot, Aurore Fleurie, Claudia Lannsjö, Anne-Claire Lukaszewicz, Johan Mårtensson, Tài Pham, Nicolas De Prost, Jean-Damien Ricard, Mervyn Singer, Gabriel Terraz, Jean-François Timsit, Christian Unge, Antoine Vieillard-Baron, Rebecka Rubenson Wahlin, Jean-François Llitjos, Anthony C. Gordon

**Affiliations:** 1 Division of Anaesthetics, Pain Medicine and Intensive Care, Imperial College London, London, UK; 2 bioMérieux, Lyon, France; 3 EA 7426 Pathophysiology of Injury-Induced Immunosuppression, Université Claude Bernard Lyon 1 – Hospices Civils de Lyon - bioMérieux, Lyon, France; 4 Assistance Publique – Hôpitaux de Paris, Hôpital Cochin, DMU Réanimation-Urgences, Service de Médecine Intensive Réanimation; Institut Cochin, INSERM U1016, CNRS UMR8104, Université Paris Cité, Paris, France; 5 Department of Infectious Diseases, Karolinska University Hospital, Stockholm, Sweden; 6 Department of Medicine Huddinge, Karolinska Institutet, Stockholm, Sweden; 7 Division of Critical Care University College London Hospitals and NIHR University College London Hospitals Biomedical Research Centre, London, UK; 8 Södersjukhuset Hospital, Stockholm, Sweden; 9 Inserm, Innovative Therapies in Haemostasis, Université Paris Cité, 75006 Paris, France; 10 Service de Médecine Intensive Réanimation, AP-HP, Hôpital Européen Georges Pompidou, 75015 Paris, France; 11 Assistance Publique – Hôpitaux de Paris, Hôpital Tenon, DMU APPROCHES, Service de Médecine Intensive Réanimation; Centre de Recherche Saint-Antoine UMRS_938 INSERM, Sorbonne Université, Paris, France; 12 Department of Perioperative Medicine and Intensive Care, Karolinska University Hospital Huddinge, Stockholm, Sweden; 13 Hospices Civils de Lyon, Lyon, France; 14 Department of Perioperative Medicine and Intensive Care, Karolinska University Hospital, Stockholm, Sweden; 15 AP-HP, Hôpital de Bicêtre, DMU CORREVE, Service de Médecine Intensive-Réanimation, FHU SEPSIS, Groupe de Recherche Clinique CARMAS, Université Paris-Saclay, Le Kremlin-Bicêtre, France; 16 Inserm U1018, Equipe d’Epidémiologie Respiratoire Intégrative, Centre de Recherche en Epidémiologie et Santé des Populations, Université Paris-Saclay, UVSQ, Univ. Paris-Sud, Villejuif, France; 17 AP-HP, GHU Henri Mondor, DMU Médecine, Service de Médecine Intensive Réanimation, IMRB, INSERM U955, Université Paris Est Créteil, Créteil, France; 18 Assistance Publique – Hôpitaux de Paris, Hôpital Louis Mourier, DMU ESPRIT, Service de Médecine Intensive Réanimation, Université Paris Cité, IAME, UMR 1137, INSERM, Colombes, France; 19 Bloomsbury Institute of Intensive Care Medicine, University College London, London, UK; 20 AP-HP, Bichat Hospital, Medical and Infectious Diseases ICU (Mi2), 75018 Paris, France; 21 IAME, INSERM, Université Paris-Cité, 75018 Paris, France; 22 Danderyds Hospital, Stockholm, Sweden; 23 Medical and Surgical ICU, University Hospital Ambroise Pare, GHU Paris-Saclay, APHP, Université Versailles Saint Quentin en Yvelines, CESP, UMR1018, Boulogne, Paris, France

**Keywords:** Sepsis, Gene-expression, Transcriptomics, Prospective study

## Abstract

**Background:**

Sepsis gene-expression sub-phenotypes with prognostic and theranostic potential have been discovered. These have been identified retrospectively and have not been translated to methods that could be deployed at the bedside. We aimed to identify subgroups of septic patients at high-risk of poor outcome, using a rapid, multiplex RNA-based test.

**Methods:**

Adults with sepsis, in the intensive care unit (ICU) were recruited from 17 sites in the United Kingdom, Sweden and France. Blood was collected at days 2–5 (S1), 6–8 (S2) and 13–15 (S3) after ICU admission and analyzed centrally. Patients were assigned into ‘high’ and ‘low’ risk groups using two models previously developed for the Immune-Profiling Panel prototype on the bioMérieux FilmArray® system.

**Results:**

357 patients were recruited (March 2021–November 2022). 69% were male with a median age of 67 years, APACHE II score of 21 and a 30% 90-day mortality rate. The proportions of high-risk patients decreased over the three sampling times (model 1: 53%, 40%, 15% and model 2: 81%, 74%, 37%). In model 1, 90-day mortality was higher in a high-risk group at each time (S1: 35% vs 24%, *p* = 0.04; S2: 43% vs 20%, *p* < 0.001; S3: 52% vs 24%, *p* = 0.007). In model 2, mortality was only significantly different at the second sampling time (S1: 30% vs 27%, *p* = 0.77; S2: 34% vs 14%, *p* = 0.002; S3: 35% vs 23%, *p* = 0.13).

**Conclusions:**

Gene-expression diagnostics can identify patients with sepsis at high-risk of poor outcomes and could be used to identify patients for precision medicine trials.

**Registration:**

ISRCTN11364482 Registered 24th September 2020.

**Supplementary Information:**

The online version contains supplementary material available at 10.1186/s13054-025-05319-5.

## Introduction

Sepsis is a heterogenous syndrome, which contributes to the difficulty in developing treatments [1]. With nearly 11 million deaths a year worldwide [2], overcoming this heterogeneity is a research priority [3, 4]. Gene-expression sepsis sub-phenotypes could help identify patients at higher risk of death [5–7] and detect treatment responsive subgroups [8, 9]. This has been done retrospectively using techniques that cannot be deployed in the intensive care unit (ICU). To influence treatment, ways of assigning patients to meaningful gene-expression groups rapidly, using small sets of genes and devices that are easy to operate are needed.

One possible solution is the FilmArray® device which is an FDA and CE-IVD certified multiplex polymerase chain reaction (PCR) system requiring five minutes of sample preparation and providing results in less than one hour from patient’s whole blood. Different gene panels can be assayed in the same FilmArray® device. The Reanimation Low Immune Status Markers (REALISM) study [10] found an informative prototype Immune Profiling Panel (IPP) [11] of genes associated with immune function and inflammation could be measured on the FilmArray® with results that were reproducible, comparable to those obtained from standard quantitative PCR and able to account for variations in cell counts and technical variability [11]. From the IPP prototype two classification models have been developed using the same eleven genes, one that predicts patients with low monocyte Human Leukocyte Antigen-DR (HLA-DR) levels (the mHLA-DR model) [12], a marker of sepsis induced immunosuppression and worse outcome [13, 14], and another that identifies patients at high-risk of death or hospital acquired infection (HAI) (the clinical worsening model) [15]. In both models, the genes CD3D, CD74, CIITA, CTLA4, CX3CR1, IFNγ, and TAP2, which are involved in processes including T-cell function and antigen presentation, were down-regulated in the high-risk groups, whereas C3AR1, CD177, IL1R2, and S100A9, involved in neutrophil function and modulation of circulating inflammatory mediators, were up-regulated [12, 15].

We hypothesized that subgroups of patients with sepsis who have gene-expression patterns suggestive of immunosuppression that result in high mortality and HAI rates could be identified by measuring this small panel of gene transcripts using a device that could be used by clinical teams, the FilmArray®. Such tools are a prerequisite for the development of gene-expression guided personalized medicine trials in sepsis. To address this, we performed an observational study to test if we could identify a subgroup of patients with sepsis at high-risk of a poor outcome using the IPP prototype. Some preliminary findings have been presented as an abstract [16].

## Methods

### Study design and participants

This was a prospective observational study conducted in 17 hospitals in the United Kingdom (4 sites), France (9 sites) and Sweden (4 sites) between March 2021 and November 2022. Patients were recruited from wards capable of providing organ support, either high-dependency units or ICUs, hence forth referred to as ICUs. The study gained ethical approval in each participating country (supplementary methods). Written informed consent was provided by participants or their legal representatives following local procedures.

We included adult patients (≥ 18 years) being treated in ICU for suspected sepsis, between 48 and 120 h after ICU admission. Eligible patients had to have received organ support for at least 24h and had to be expected to require ongoing critical/high dependency care for at least one more calendar day. Exclusion criteria included immunosuppression due to causes other than sepsis and patients with either a ‘withdrawal of life-sustaining treatment’ decision or who were not expected to survive 24 h. Full details can be found in the supplement.

### Study sample collection and assignment to gene-expression sub-groups

Blood samples were collected at enrolment into the study (day 2–5 after ICU admission, S1), on day 6–8 (48-96h after the first sample, S2) and day 13–15 after ICU admission (S3), supplementary figure S1. Sampling times were based on data describing the optimal timing of IPP analysis [10–12]. 2.5 mls of blood were collected into PAXgene tubes and left at room temperature for at least 2h before being frozen at −20°C or −80°C. Samples were sent centrally to bioMérieux for measurement of gene-expression with the IPP prototype on the FilmArray® system.

Samples were assigned into ‘low’ and ‘high-risk’ groups based on two previously described models using the same set of eleven genes (supplementary table S1) measured on the FilmArray® device. The first was optimized to predict either death or HAI (clinical worsening model) [15]. The second was optimized to predict patients with a monocyte HLA-DR (mHLA-DR model) less than 8,000 antibodies per monocyte [12]. Patients were assigned to these groups using only gene-expression data and did not include clinical or outcome data. A probability threshold of 0.5 was used to define group membership. Patient’s IPP classifications were not provided to clinical teams.

### Outcome measures

Key outcome measures were 90-day all-cause mortality and new HAI up until hospital discharge or day-90. We took a two-step approach to define HAI. First, local investigators reported HAI defined as any new episode of antimicrobials (excluding prophylactic antibiotics) started at least 48h after a previous course was stopped. This definition was intended to have a high sensitivity to detect HAI. These potential HAIs were reviewed by an adjudication panel, three clinicians independent of the recruiting hospital, who judged if an HAI was either definite or possible based on modified European Centre for Disease Prevention and Control criteria [17, 18]. This approach was designed to identify patients with a high certainty of infection.

Secondary outcome measures are detailed in the supplement.

### Sample size and statistical analysis

From data from the REALISM study [18] we predicted 40% of recruited patients would be in the ‘high-risk’ group and would have a mortality rate of 32% compared to 20% in the ‘low-risk’ group (overall cohort mortality 25%) meaning 577 patients would provide 90% power to detect this difference between groups with an alpha of 0.05. To account for potential loss to follow-up we aimed to recruit 600 patients.

Descriptive statistics characterizing the study participants are presented as medians and interquartile ranges for continuous variables and as counts and percentages for categorical variables. Statistical comparisons were made with Pearson’s Chi-squared test or exact Fisher test for categorical variables or Analysis of Variance, Wilcoxon test or t-test for continuous data as appropriate. Analysis of HAI was done by categorizing patients into those who had no event, those who died without an HAI and those with an HAI to take account of the competing risk of death with HAI. All HAI data were censored meaning that analysis was restricted to HAIs that occurred after sample collection with exclusion of patients in whom HAI occurred prior to IPP sampling. Only the first incidence of HAI was considered. Logistic regression, controlling for the need for organ support at the time of sample collection, was used to determine if associations between gene-expression groups and mortality were independent of the need for organ support. Statistical analysis was conducted with R software v4.1.3 [19].

## Results

**Fig. 1:**
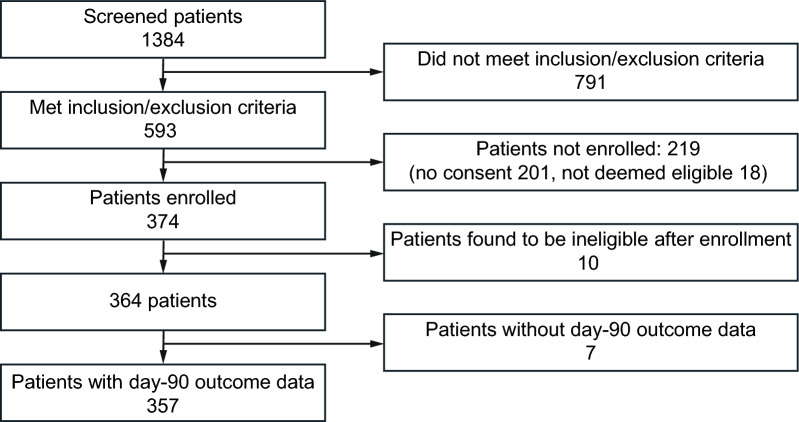
Consort diagram showing the number of patients finally included in the study

Due to the COVID-19 pandemic the study was delayed, and recruitment was slower than predicted. Recruitment terminated prior to reaching the planned sample size when study funding terminated. In total 374 patients were enrolled, of whom 10 were excluded as it became apparent after recruitment but prior to analysis that they failed to meet the inclusion and exclusion criteria, and 7 were lost to follow-up (Fig. 1) leaving 357 patients for analysis.

Patients were recruited across the UK, France and Sweden (supplementary table S2). Baseline characteristics and outcome by country are shown in Tables 1 and Supplementary Tables S3, S4. Patients were recruited a median of 3 days (inter quartile range (IQR) 3–4 days) after admission to ICU. The median age was 67 years, 69% were male and the median APACHE II score was 21. Most admissions were medical (73%) with lung (53%) being the commonest infection site. Overall, 90-day mortality was 30%. A new course of antibiotics was commenced in 27% of patients and HAI was confirmed as either definite or possible via the adjudication panel in 15%, the most common site of HAI was the respiratory tract (Supplementary table S5). 357 patients had samples collected at the first (day 2–5), 303 at the second (day 6–8) and 169 at the final (day 13–15) time point. The first sample was collected a median of 3 (IQR 3–4) days after ICU admission, the second 6 (IQR 5–7) days and the third 15 (IQR 14–17) days.

**Table 1:** Demographic, clinical features at enrolment and outcomes of recruited participants by country

	France	Sweden	United Kingdom	Global population
n	88	57	212	357
Number of males	63 (72)	38 (67)	146 (69)	247 (69)
Age (years)	69 (59–75)	69 (59–74)	64 (53–73)	67 (56–74)
Number of Caucasians	-	54 (96)	126 (59)	180/*268* (67)
BMI (kg/m^2^)	26 (23–30) *n* = *76*	27 (25–32) *n* = *57*	26 (23–29) *n* = *202*	26 (23–30) *n* = *335*
Medical Admission	80 (91)	41 (72)	138 (65)	259 (72)
Charlson comorbidity score	3 (2–5)	3 (2–4)	3 (1–4)	3 (1–4)
One or more comorbidity	64 (73)	31 (54)	120 (57)	215 (60)
*Source of infection*				
n	*87*	*57*	*212*	*356*
Lung	41 (47)	25 (44)	123 (58)	189 (53)
Abdomen	16 (18)	10 (17)	27 (13)	53 (15)
Soft tissue or line	5 (6)	5 (9)	16 (8)	26 (7)
Urine	10 (11)	3 (5)	13 (6)	26 (7)
Neurological	1 (1)	3 (5)	12 (6)	16 (5)
Primary bacteremia	3 (3)	2 (4)	3 (1)	8 (2)
Other	11 (13)	9 (16)	18 (9)	38 (11)
*Baseline disease severity, clinical parameters and organ support*				
SOFA score	9.5 (8.0–12.0) *n* = *74*	9.0 (7.0–11.0) *n* = *50*	9.0 (7.0–11.0) *n* = *196*	9.0 (7.0–11.0) *n* = *320*
APACHE II score	24 (18–30) *n* = *73*	21 (17–26) *n* = *54*	21 (17–26) *n* = *196*	21 (17–26) *n* = *323*
Lactate (mmol/L)	1.4 (1.0–2.5) *n* = *85*	1.1 (0.9–1.5) *n* = *56*	1.2 (0.9–1.7) *n* = *211*	1.2 (0.9–1.8) *n* = *352*
Creatinine (μmol/L)	131 (79–227) *n* = *88*	100 (67–197) *n* = *57*	80 (60–137) *n* = *210*	90 (64–165) *n* = *355*
Bilirubin (μmol/L)	12 (7–24) *n* = *81*	13 (8–30) *n* = *52*	10 (6–19) *n* = *211*	11 (7–21) *n* = *344*
Platelets (× 10^9^/L)	168.0 (83.5–324.5) *n* = *83*	197.5 (135.0–271.5) *n* = *56*	184.0 (123.0–289.0) *n* = *207*	185.0 (113.0–290.0) *n* = *346*
PaO_2_/FiO_2_ (mmHg)	202 (134–278) *n* = *82*	218 (164–271) *n* = *56*	204 (143–266) *n* = *209*	208 (143–269) *n* = *347*
Mechanical ventilation	69 (78)	37 (65)	150 (71)	256 (72)
Acute Kidney Injury	45 (51) *n* = *88*	21 (37) *n* = *57*	62 (29) *n* = *211*	128 (36) *n* = *356*
*Outcomes*				
Day-90 mortality	38 (43)	11 (19)	57 (27)	106 (30)

Categorical variables are given as number (percentage) and continuous variables as median (interquartile range), where the denominator for categorical variables differs from the total population it is given in italics in the relevant section

*BMI* body mass index, *SOFA* sequential organ failure assessment, *APACHE* acute physiology and chronic health evaluation

### Clinical worsening model

For the model developed to predict death or HAI known as the ‘clinical worsening model’ gene-expression differences between the ‘high-risk’ and ‘low-risk’ groups reflected those seen in model development [15] (Supplementary table S1, figure S2). Genes associated with T-cell function, leukocyte binding and adhesion and antigen presentation had relatively lower expression in the ‘high’ versus ‘low-risk’ group whilst those associated with neutrophil activation and accumulation had higher expression.

**Fig. 2:**
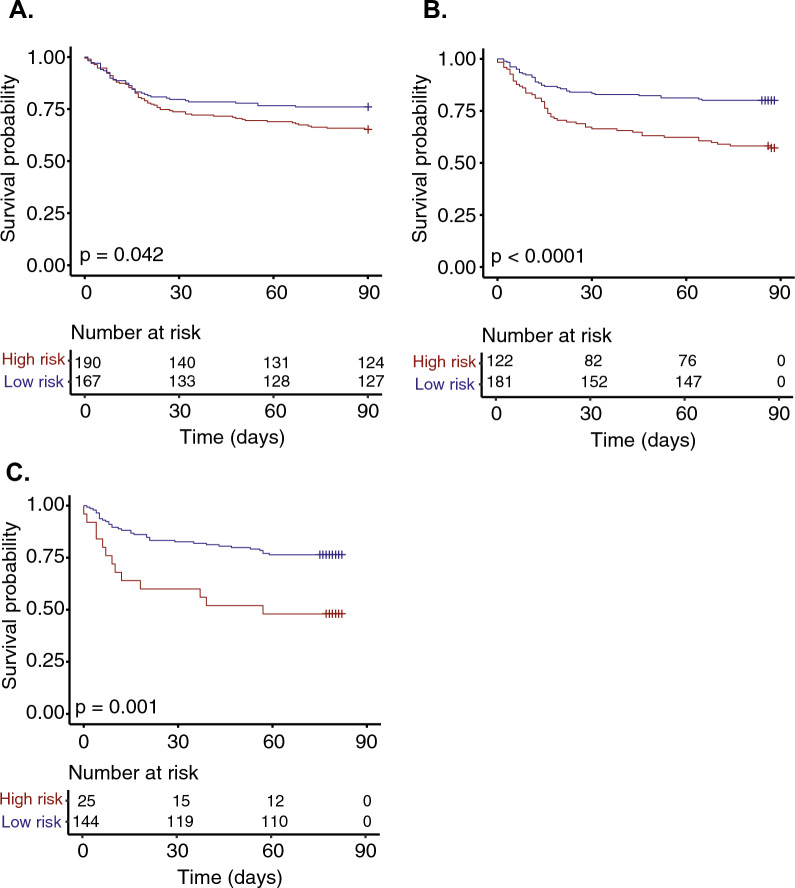
Kaplan–Meier curves for survival by gene-expression groups based on the clinical worsening model. **A** Sample S1 (day 2–5), **B** sample S2 (day 6–8) and C) sample S3 (day 13–15). The ‘high-risk’ group is shown in red and the ‘low-risk’ group in blue. For the S2 and S3 plots time 0 is the time of sample collection and crosses represent censoring due to left shift of the data. *p*-values are given from the log-rank test

**Fig. 3:**
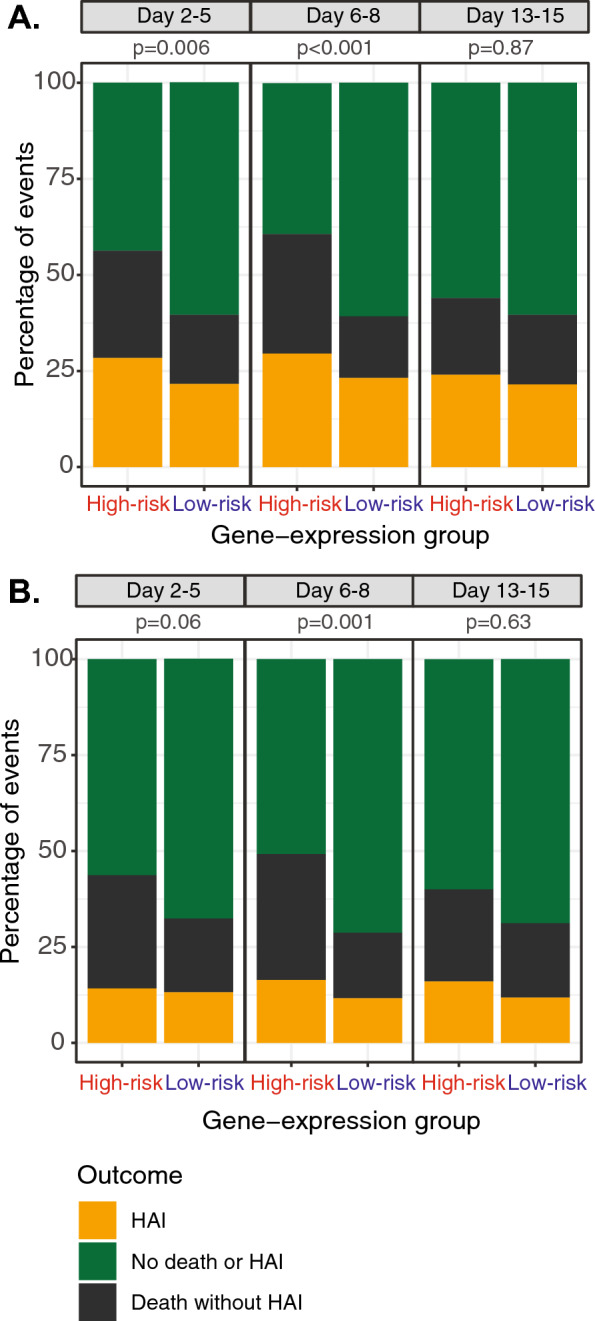
Hospital acquired infection (HAI) events by day-90. At each time point HAI data was censored for HAIs that occurred prior to sampling, with HAI events representing those occurring after the IPP sampling (yellow) shown compared to death without HAI (black) as a competing risk and patients with neither event (green). HAI was assessed as **A** a new course of antibiotics after at least 48h without antibiotics and **B** of those patients the ones judged to either definitely or possibly have HAI by an independent panel

The proportion of patients in the ‘high-risk’ group decreased over time (day 2–5 190 patients (53%), day 6–8 122 (40%), day 13–15 25 (15%), supplementary figure S3). At all sampling time points the ‘high-risk’ group had significantly higher day-90 mortality (day 2–5 35% vs 24%, *p* = 0.04; day 6–8 43% vs 20%, *p* < 0.001; day 13–15 52% vs 24% *p* = 0.007), Fig. 2 and Table 2. HAI rates, including death as a competing risk, were significantly different between risk-groups on days 2–5 and 6–8 (Fig. 3) with higher proportions of patients in the ‘low-risk’ group having uncomplicated recoveries, neither dying nor acquiring HAI (Fig. 3, Table 2). When HAI rates were directly compared there were no significant difference at any time between groups (Table 2).

**Table 2:** Key outcomes by sampling period for the clinical worsening model

	Sample S1 (Day 2–5)			Sample S2 (Day 6–8)			Sample S3 (Day 13–15)		
	High-risk	Low-risk	*p*-value	High-risk	Low-risk	*p*-value	High-risk	Low-risk	*p*-value
n	190	167	**-**	122	181	-	25	144	-
Death									
Day-90	66 (35)	40 (24)	**0.04**	52 (43)	36 (20)	** < 0.001**	13 (52)	34 (24)	**0.007**
Hospital acquired infection (HAI)									
New course of antibiotics									
Day-90			**0.01**			** < 0.001**			0.34
No event	81/188 (43)	95/161 (59)		42/116 (36)	104/175 (59)		6/17 (35)	66/123 (54)	
Death with no HAI	53/188 (28)	30/161 (19)		38/116 (33)	29/175 (17)		5/17 (29)	26/123 (21)	
HAI	54/188 (29)	36/161 (22)		36/116 (31)	42/175 (24)		6/17 (35)	31/123 (25)	
Day-90 HAI censored	54/188 (29)	36/161 (22)	0.22	36/116 (31)	42/175 (24)	0.23	6/17 (35)	31/123 (25)	0.39
Adjudicated									
Day-90			0.07			** < 0.001**			0.13
No event	105/188 (56)	109/163 (67)		56/116 (48)	126/178 (71)		8/18 (44)	91/136 (67)	
Death with no HAI	56/188 (30)	32/163 (20)		40/116 (35)	31/178 (17)		6/18 (33)	28/136 (21)	
HAI	27/188 (14)	22/163 (14)		20/116 (17)	21/178 (12)		4/18 (22)	17/136 (13)	
Day-90 HAI censored	27/188 (14)	22/163 (14)	0.94	20/116 (17)	21/178 (12)	0.25	4/18 (22)	17/136 (13)	0.27

*p*-values are given from chi-squared testing, those in bold are significant at *p* < 0.05

At all times patients in the ‘low-risk’ group had a greater number of ventilator, renal replacement therapy, vasopressor, and ICU-free days compared to the high-risk group (supplementary table S6) but without difference in day-90 health-related quality of life (supplementary table S7).

Patients in the ‘high-risk’ group were less likely to have a respiratory source of their initial infection and more likely to have an abdominal source than those in the ‘low-risk’ group (Table 3, supplementary table S8, supplementary figure S4). Although disease characteristics were often worse in patients in the ‘high-risk’ group, for example APACHE II score, lactate, creatinine and bilirubin (Table 3, supplementary table S8), differences were often small. For example, differences between APACHE II and lactate at baseline between patients in the ‘high’ and ‘low-risk’ group were 22 (18–27) vs 21 (16–26), *p* = 0.02 and 1.3 (1.0–1.9)mmol/L vs 1.0 (0.8–1.3)mmol/L, p < 0.001 respectively. Although patients in the ‘high-risk’ group were more likely to require organ support than those in the ‘low-risk’ group (Table 3, supplementary table S8), when mortality analysis was adjusted for the need for invasive mechanical ventilation, renal replacement therapy and vasopressors ‘high-risk’ patients remained at increased risk of 90-day mortality (day 2–5: odds ratio (OR) 1.62 (95% confidence interval: 1.00–2.66), *p* = 0.05; day 6–8: 2.71 (1.60–4.63), p = 0.0002; day 13–15: 2.94 (1.09–7.93), *p* = 0.03). No clinical parameters performed well at predicting membership of the ‘high-risk’ group (supplementary figure S5).

**Table 3:** Baseline characteristics of the gene-expression groups at the first sampling time point (day 2–5)

	High-risk	Low-risk	*p*-value
n	190	167	–
Number of males	134 (71)	113 (68)	0.64
Age (years)	67 (57–75)	67 (54–74)	0.42
Ethnicity			0.10
n	*133*	*135*	
Caucasian	92 (69)	88 (65)	
Other	20 (15)	28 (21)	
Asian	16 (12)	8 (6)	
Black	5 (4)	11 (8)	
BMI (kg/m^2^)	25.9 (22.9–28.5)	27.1 (23.7–31.2)	**0.01**
Admission type			0.66
Medical	134 (71)	125 (75)	
Emergency surgery	44 (23)	33 (20)	
Elective surgery	12 (6)	9 (5)	
Charlson comorbidity score	3.0 (2.0–4.0)	3.0 (1.0–5.0)	0.87
Source of infection			** < 0.001**
n	189	167	
Lung	83 (44)	106 (64)	
Abdomen	43 (23)	10 (6)	
Soft tissue or line	13 (7)	13 (8)	
Urine	14 (7)	12 (7)	
Neurological	6 (3)	10(6)	
Primary bacteraemia	5 (3)	3 (2)	
Other	25 (13)	13 (8)	
SOFA score	10.0 (7.0–12.0)	7.5 (6.0–9.8)	** < 0.001**
APACHE II score	22.0 (18.0–27.0)	21.0 (16.0–26.0)	**0.02**
Invasive mechanical ventilation, n/N (%)	146/187 (78)	101/159 (64)	**0.004**
RRT, n/N (%)	43/187 (23)	16/159 (10)	**0.002**
Vasopressors, n/N (%)	129/187 (69)	99/159 (62)	0.23

Categorical variables are given as number (percentage) and continuous variables as median (interquartile range), where the denominator for categorical variables differs from the total population it is given in italics in the relevant section. (BMI, body mass index; SOFA, sequential organ failure assessment; APACHE, acute physiology and chronic health evaluation; RRT renal replacement therapy) p-values in bold are those that were significant at <0.05

### mHLA-DR model

Overall, we found that the model designed to predict risk based on mHLA-DR performed poorly at identifying a group of patients at higher risk of death (supplementary figure S3, supplementary table S9, S10, S11).

### Risk group trajectory

Patients moved between the ‘high’ and ‘low’ risk groups in both models with the highest mortality being in those who stayed or transitioned into the ‘high’ risk group (supplementary figures S3, S6, table S12). When analysis was restricted to patients with samples available at both day 2–5 and day 6–8, patients who remined in the ‘high-risk’ group had the fewest ventilation, renal replacement therapy, vasopressor, ICU and hospital free days.

## Discussion

We demonstrated that patients with sepsis can be categorized into gene-expression sub-groups that have different outcomes based on a small panel of genes measured on a commercially available device, already available in hospitals. A ‘high-risk’ group with gene-expression features of immunosuppression including altered T-cell function and antigen presentation were at high risk of mortality and had an increased use of ICU resources.

We tested two risk prediction models derived using the IPP genes, one that predicted patients expected to have low mHLA-DR and another that predicted clinical risk. Of the models the clinical worsening model consistently identified a group at higher risk of death at 90-days whereas the mHLA-DR model did not. This could be for several reasons, first we did not have mHLA-DR measurements available so, although this model has been previously validated [12], we do not know how well it estimates this parameter in our population. Secondly, the clinical worsening model was developed specifically to predict clinical deterioration rather than low mHLA-DR and therefore it is perhaps not surprising that it appears be the better predictor of clinical deterioration rather than a proxy risk predictor.

Although we found clinical differences between the sub-groups, clinical parameters performed poorly at predicting IPP group membership suggesting that the immunological information provided by the IPP supplements routine clinical data and provides another indicator of risk not captured by other tests. The IPP genes give some insight into the underlying immunobiology of the two sub-groups. The genes upregulated in the ‘high-risk’ group suggest neutrophil dysfunction whilst those that are downregulated predict a reduction in T-cells and antigen presentation [11, 12, 15]. The patterns of gene-expression between the IPP groups are similar to those previously reported between the sepsis response signature (SRS) 1 and SRS2 sub-phenotypes [5, 6] suggesting possible similarities between the two profiling methods.

We hypothesized that increased mortality in ‘high-risk’ patients would be a consequence of an increase in HAI due to immunosuppression. However, this was not seen. It may be that an inability to clear the initial infection leads to mortality in this group, this is supported by findings that 89% of patients that died from septic shock had persistent evidence of infection at postmortem [20]. If patients die from the unresolved primary infection, they will never be able to contract a new secondary HAI. The finding that death without HAI was higher in the ‘high-risk groups’ supports this. Similarly, clinically identifiable infections may not be the main driver of mortality. Viral reactivation is more common in septic patients with immunosuppressed gene-expression sub-phenotypes [21] and it may be that this is responsible for a persistent cycle of inflammation and organ failure that contributes to death in ‘high-risk’ patients.

Although identifying patients at elevated risk may have utility, the real benefit of gene-expression sub-groups will be if they allow more targeted treatment. The demonstration that host gene-expression can be measured on a device that could be used in the clinical environment and that it can identify a group at risk of a poor outcome supports the development of a personalized approach to sepsis. This could be by investigating therapies predicted to benefit the ‘high-risk’ IPP group, for example, GM-CSF which boosts antigen presentation [22] or recombinant human interleukin-7 which increases CD4 lymphocyte proliferation [23] and IFNγ producing T-cells [24]. Corticosteroids have been shown to have a differential treatment effect based on SRS endotypes [8]. As patterns of gene-expression show some similarities between the SRS sub-phenotypes and the groups reported here, this may be a viable method to stratify patients to this intervention. Alternatively, the technology tested here could be repurposed to allow assignment into other gene-expression sub-phenotypes.

Moving away from syndromic diagnoses, such as sepsis and acute respiratory distress syndrome, to identifying pathobiological processes responsive to specific treatments, known as treatable traits, is an important step towards precision medicine in critical illness [4, 25]. However, for these to be deployed clinically, novel diagnostics are required that can identify these subgroups of patients. This study demonstrates that this could be possible using the FilmArray® device. Such precision medicine trials would require that such tests can be shown to be used in a local laboratory or even in a near-patient environment by clinical staff. Although that was not possible in this study due to the COVID-19 restrictions, the pandemic has subsequently accelerated the adoption of rapid PCR based diagnostics. The FilmArray® device has been successfully used in both adult and paediatric ICUs as a point of care test to guide antibiotic management for hospital-acquired and ventilator associated pneumonia [26].

It is likely that such biomarker guided precision medicine clinical trials would initially be used to simply select patients for immunomodulation therapies. The fact that the signal for increased mortality appears to become even more marked the longer the patient remain in the high-risk group opens the possibility that such biomarkers could be used to monitor response to therapy and guide when such immune modulating treatments could be stopped. However, this will require greater understanding of gene expression profiles over time and particularly their response to immune modulating therapy. Further studies will be needed to understand how gene-expression sub-phenotypes perform earlier in sepsis than studied here, for example at ICU admission or in the emergency department, and to validate the clinical applicability of the longitudinal dynamics of gene-expression group membership.

Although this study has several strengths, for example its prospective, international multi-center design and its well-defined approach to diagnosing HAI using internationally recognized criteria, it also has limitations. The study failed to meet its recruitment target due to the impact of the COVID-19 pandemic. This reduction in power may account for why some findings, importantly including the incidence of HAI, failed to reach statistical significance (i.e. could be a false negative result) and inevitably provides less precision around the size of mortality differences. However, despite this we were still able to detect clinically useful, statistically significant differences between clinical risk groups.

The mHLA-DR risk model did not reach statistical significance and this may reflect the reduced power of the reduced sample size. Without mHLA-DR measurements we cannot confirm if it predicted low mHLA-DR values as seen in previous validation studies [12] so without direct validation the results must be interpreted cautiously.

Due to limitations of clinical tests, including the accuracy of microbiological testing, it is often difficult to diagnose HAI with certainty. Our approach of using two definitions, one that would capture the broadest definition of HAI based on “bedside” clinical decision making and the other using an independent panel to limit cases to only those most likely to be genuine HAIs based on predefined criteria was designed to overcome this challenge.

In this study we have taken a categorical approach to phenotyping, as has been done by many studies in the field [5–7, 27] and have managed to demonstrate that this is able to determine clinically useful groups. However, treating the strength of group membership as a continuous trait may be more beneficial, providing more granular classification, avoiding issues around borderline cases and recognizing that critical illness induced immunosuppression is not binary. The evaluation of this approach is beyond the scope of this study. Finally, although samples were processed using the IPP pouches on the FilmArray® system, due to a lack of available devices (due to supply issues during the pandemic) this was done centrally and not in the clinical environment. Future studies will need to assess the utility of these gene-expression group allocations when devices are deployed near to the patient, this would also serve to provide further validation of the test in other cohorts. However, there is wealth of experience using the FilmArray® technology in hospitals for pathogen detection which demonstrates the device’s ease of use and accuracy.

In conclusion we demonstrated that subgroups of patients with sepsis with different outcomes can be identified using a small set of gene expression transcripts measured on a device that requires minimal sample handling and with a rapid turnaround time. This supports the feasibility of using gene-expression groups clinically, for example to stratify patients into clinical trials of immune modulating therapies to deliver a precision medicine approach to sepsis care.

## Supplementary Information

Additional file 1

## Data Availability

Individual participant data that underlie the results in this article, after de-identification (text, table, and figures) will be made available from the corresponding author on submission of a data request application, including scientific rationale and intended use statements. Applications will be reviewed by the Consortium steering committee and are subject to data sharing agreements.

## References

[1] Marshall JC. Why have clinical trials in sepsis failed? Trends Mol Med. 2014;20(4):195–203. 24581450 10.1016/j.molmed.2014.01.007

[2] Rudd KE, Johnson SC, Agesa KM, Shackelford KA, Tsoi D, Kievlan DR, et al. Global, regional, and national sepsis incidence and mortality, 1990–2017: analysis for the Global Burden of Disease Study. Lancet. 2020;395(10219):200–11. 31954465 10.1016/S0140-6736(19)32989-7 PMC6970225

[3] Coopersmith CM, De Backer D, Deutschman CS, Ferrer R, Lat I, Machado FR, et al. Surviving sepsis campaign: research priorities for sepsis and septic shock. Intensive Care Med. 2018;44(9):1400–26. 29971592 10.1007/s00134-018-5175-z PMC7095388

[4] Gordon AC, Alipanah-Lechner N, Bos LD, Dianti J, Diaz JV, Finfer S, et al. From ICU syndromes to ICU subphenotypes: consensus report and recommendations for developing precision medicine in ICU. Am J Respir Crit Care Med. 2024;2(10):155–66. 10.1164/rccm.202311-2086SO PMC11273306 38687499

[5] Burnham KL, Davenport EE, Radhakrishnan J, Humburg P, Gordon AC, Hutton P, et al. Shared and distinct aspects of the sepsis transcriptomic response to fecal peritonitis and pneumonia. Am J Respir Crit Care Med. 2017;196(3):328–39. 28036233 10.1164/rccm.201608-1685OC PMC5549866

[6] Davenport EE, Burnham KL, Radhakrishnan J, Humburg P, Hutton P, Mills TC, et al. Genomic landscape of the individual host response and outcomes in sepsis: a prospective cohort study. Lancet Respir Med. 2016;4(4):259–71. 26917434 10.1016/S2213-2600(16)00046-1 PMC4820667

[7] Scicluna BP, van Vught LA, Zwinderman AH, Wiewel MA, Davenport EE, Burnham KL, et al. Classification of patients with sepsis according to blood genomic endotype: a prospective cohort study. Lancet Respir Med. 2017;5(10):816–26. 28864056 10.1016/S2213-2600(17)30294-1

[8] Antcliffe DB, Burnham KL, Al-Beidh F, Santhakumaran S, Brett SJ, Hinds CJ, et al. Transcriptomic signatures in sepsis and a differential response to steroids from the VANISH randomized trial. Am J Respir Crit Care Med. 2019;199(8):980–6. 30365341 10.1164/rccm.201807-1419OC PMC6467319

[9] Wong HR, Cvijanovich NZ, Anas N, Allen GL, Thomas NJ, Bigham MT, et al. Developing a clinically feasible personalized medicine approach to pediatric septic shock. Am J Respir Crit Care Med. 2015;191(3):309–15. 25489881 10.1164/rccm.201410-1864OC PMC4351580

[10] Venet F, Textoris J, Blein S, Rol ML, Bodinier M, Canard B, et al. Immune profiling demonstrates a common immune signature of delayed acquired immunodeficiency in patients with various etiologies of severe injury. Crit Care Med. 2022;50(4):565–75. 34534131 10.1097/CCM.0000000000005270

[11] Tawfik DM, Vachot L, Bocquet A, Venet F, Rimmelé T, Monneret G, et al. Immune profiling panel: a proof-of-concept study of a new multiplex molecular tool to assess the immune status of critically ill patients. J Infect Dis. 2020;222(Suppl 2):S84. 32691839 10.1093/infdis/jiaa248 PMC7372218

[12] Peronnet E, Blein S, Venet F, Cerrato E, Fleurie A, Llitjos JF, et al. Immune profiling panel gene set identifies critically ill patients with low monocyte human leukocyte antigen-DR expression: preliminary results from the reanimation low immune status marker (REALISM) Study. Crit Care Med. 2023;51(6):808. 36917594 10.1097/CCM.0000000000005832 PMC10187625

[13] Tremblay JA, Peron F, Kreitmann L, Textoris J, Brengel-Pesce K, Lukaszewicz AC, et al. A stratification strategy to predict secondary infection in critical illness-induced immune dysfunction: the REALIST score. Ann Intensive Care. 2022;12:76. 35976460 10.1186/s13613-022-01051-3 PMC9382015

[14] De Roquetaillade C, Dupuis C, Faivre V, Lukaszewicz AC, Brumpt C, Payen D. Monitoring of circulating monocyte HLA-DR expression in a large cohort of intensive care patients: relation with secondary infections. Ann Intensive Care. 2022;12:39. 35526199 10.1186/s13613-022-01010-y PMC9079217

[15] Peronnet E, Terraz G, Cerrato E, Imhoff K, Blein S, Brengel-Pesce K, et al. Use of immune profiling panel to assess the immune response of septic patients for prediction of worsening as a composite endpoint. Sci Rep. 2024;14(1):1–10. 38760488 10.1038/s41598-024-62202-z PMC11101454

[16] Antcliffe DB, Peronnet E, Pène F, Strålin K, Brealey D, Cleaver R, et al. An International Multicenter Prospective Observational Clinical Study to Identify Gene-expression Sepsis Immune Subgroups and Their Associations With Clinical Outcomes. American Thoracic Society International Conference Meetings Abstracts. 2024;A4992–A4992.

[17] Point prevalence survey of healthcare-associated infections and antimicrobial use in European acute care hospitals—protocol version 5.3 [Internet]. [cited 2024 Feb 14]. Available from: https://www.ecdc.europa.eu/en/publications-data/point-prevalence-survey-healthcare-associated-infections-and-antimicrobial-use-3

[18] Rol ML, Venet F, Rimmele T, Moucadel V, Cortez P, Quemeneur L, et al. The REAnimation low immune status markers (REALISM) project: a protocol for broad characterisation and follow-up of injury-induced immunosuppression in intensive care unit (ICU) critically ill patients. BMJ Open. 2017;7(6):15734. 10.1136/bmjopen-2016-015734 PMC5726091 28637738

[19] R Core Team. R: a language and environment for statistical computing. R Foundation for Statistical Computing; 2015.

[20] Torgersen C, Moser P, Luckner G, Mayr V, Jochberger S, Hasibeder WR, et al. Macroscopic postmortem findings in 235 surgical intensive care patients with sepsis. Anesth Analg. 2009;108(6):1841–7. 19448210 10.1213/ane.0b013e318195e11d

[21] Goh C, Burnham KL, Ansari MA, de Cesare M, Golubchik T, Hutton P, et al. Epstein-Barr virus reactivation in sepsis due to community-acquired pneumonia is associated with increased morbidity and an immunosuppressed host transcriptomic endotype. Sci Rep. 2020;10(1):1–8. 32555213 10.1038/s41598-020-66713-3 PMC7299986

[22] Mathias B, Szpila BE, Moore FA, Efron PA, Moldawer LL. A review of GM-CSF therapy in sepsis. Medicine. 2015;94(50):e2044. 26683913 10.1097/MD.0000000000002044 PMC5058885

[23] Venet F, Foray AP, Villars-Méchin A, Malcus C, Poitevin-Later F, Lepape A, et al. IL-7 Restores lymphocyte functions in septic patients. J Immunol. 2012;189(10):5073–81. 23053510 10.4049/jimmunol.1202062

[24] Thampy LK, Remy KE, Walton AH, Hong Z, Liu K, Liu R, et al. Restoration of T Cell function in multi-drug resistant bacterial sepsis after interleukin-7, anti-PD-L1, and OX-40 administration. PLoS ONE. 2018;13(6):e0199497. 29944697 10.1371/journal.pone.0199497 PMC6019671

[25] Maslove DM, Tang B, Shankar-Hari M, Lawler PR, Angus DC, Kenneth Baillie J, et al. Redefining critical illness. Nat Med. 2022;28:1141–8. 35715504 10.1038/s41591-022-01843-x

[26] Enne VI, Stirling S, Barber JA, High J, Russell C, Dhesi Z, et al. INHALE WP3, a multicentre, open-label, pragmatic randomised controlled trial assessing the impact of rapid, ICU-based, syndromic PCR, versus standard-of-care on antibiotic stewardship and clinical outcomes in hospital-acquired and ventilator associated pneumonia. Intensive Care Med. 2025;In press. 10.1007/s00134-024-07772-2 PMC11903508 39961847

[27] Calfee CS, Delucchi K, Parsons PE, Thompson BT, Ware LB, Matthay MA. Subphenotypes in acute respiratory distress syndrome: Latent class analysis of data from two randomised controlled trials. Lancet Respir Med. 2014;2(8):611–20. 24853585 10.1016/S2213-2600(14)70097-9 PMC4154544

