# Supplementary material for: An international observational study validating gene-expression sepsis immune subgroups
Source: Crit Care. 2025 Mar 3;29:98. doi: 10.1186/s13054-025-05319-5 (PMC11877781; doi:10.1186/s13054-025-05319-5)

**Supplementary Material: An International Prospective Observational Study Validating Gene-expression Sepsis Immune Subgroups.**

David B Antcliffe, Estelle Peronnet, Frédéric Pène, Kristoffer Strålin, David Brealey, Sophie Blein, Richard Cleaver, Maria Cronhjort, Jean-Luc Diehl, Guillaume Voiriot, Aurore Fleurie, Claudia Lannsjö, Anne-Claire Lukaszewicz, Johan Mårtensson, Tài Pham, Nicolas De Prost, Jean-Damien Ricard, Mervyn Singer, Gabriel Terraz, Jean-François Timsit, Christian Unge, Antoine Vieillard-Baron, Rebecka Rubenson Wahlin, Jean-François Llitjos, Anthony C Gordon

**Supplementary Methods**

**Supplementary table S1.** Immune Profiling Panel (IPP) genes.

**Supplementary table S2:** Number of patients recruited by country and site.

**Supplementary table S3:** Full list of comorbidities by recruiting country.

**Supplementary table S4:** Study outcomes by recruiting country.

**Supplementary table S5:** Source of infection for hospital acquired infections.

**Supplementary table S6:** Clinical risk-based model outcomes by sampling period.

**Supplementary table S7:** Health-related quality of life outcomes at day-90 for the clinical risk-based model.

**Supplementary table S8:** Baseline patient characteristics and clinical features at the time of sampling by risk group form the clinical deterioration model.

**Supplementary table S9:** mHLA-DR based model outcomes by sampling period.

**Supplementary table S10:** Health-related quality of life outcomes at day-90 for the mHLA-DR model.

**Supplementary table S11:** Baseline patient characteristics and clinical features at the time of sampling by risk group form the mHLA-DR model.

**Supplementary table S12:** Outcome measures by trajectory between risk groups over the first two sampling time points by the mHLA-DR and clinical worsening models.

**Supplementary Figure S1** Diagram showing the timing of patient sampling in relationship to ICU admission

**Supplementary Figure S2** Comparison of gene-expression of the IPP genes between groups in the clinical worsening and mHLA-DR models.

**Supplementary Figure S3** Proportion of patients in the high-risk group by time point.

**Supplementary Figure S4** Significant baseline characteristics by gene-expression group

**Supplementary Figure S5** Receiver operating characteristic curves showing the ability of clinical variables to predict IPP groups.

**Supplementary Figure S6** Bar plots showing trajectory between risk groups over sampling times and association of risk-group transitions with 90-day mortality.

**Supplementary Methods**

*Organisation Providing Ethical Approval*

The Health and Care Research Wales research ethics committee (20/LO/1163) in the UK; the Comité de Protection des Personnes Ile de France X in France and the Swedish Ethical Review Authority in Sweden.

*Inclusion Criteria*

Adult patients (≥18 years) admitted to ICU for between 48 and 120 hours and being (or having been) treated for suspected sepsis (requiring intravenous antibiotics to treat a known or suspected infection with acute organ dysfunction) during the ICU admission were eligible. Eligible patients had to have received organ support (any form of mechanical ventilation, non-invasive ventilation or high-flow nasal oxygen; or any intravenous continuous infusion of vasopressors or inotropes; or any form of continuous renal replacement therapy or acute and new intermittent hemodialysis with at least two episodes) for at least 24h and had to be expected to require ongoing care in an environment capable of providing organ support (e.g. an ICU or high dependency unit) for at least one more calendar day.

*Exclusion Criteria*

Severe neutropenia (neutrophil count <0.5 x 10^9^/L) due to an underlying disease or treatment but not sepsis,

Corticosteroids (intravenously or oral) of more than an equivalent dose of prednisolone 0.1mg/kg for at least 7 days within the past 30 days (not as part of sepsis management).

Use of therapeutic antibodies during this admission.

Onco-hematological disease treated within the 5 years before inclusion.

Allogenic hematopoietic stem cell transplantation.

Chemotherapy or immunotherapy within the last 36 months prior to inclusion.

Innate immune deficiency (e.g. severe combined immunodeficiency).

Acquired immune deficiency (e.g. HIV or AIDS).

Patients receiving any other immunosuppressive therapy (e.g. anti-TNF therapies).

Patients with a ‘withdrawal of life-sustaining treatment’ decision at the time of inclusion.

Patients not expected to survive 24 hours.

Participation in an interventional study of an immunomodulating therapy or any other unlicensed therapy.

Pregnant or breastfeeding women.

No social security insurance (France only).

Patients with restricted liberty, prisoners or those under legal protection.

Patients previously enrolled into the study.

*IPP gene-expression analysis*

The IPP pouches contained all the biochemical reagents and primers lyophilized and ready for use upon hydration. After sample injection (100μL of the PAXgene sample) and pouch insertion in the FilmArray, through a sequence of automated steps nucleic acids were isolated from the sample, then RNA was reversed transcribed and amplified in the first-stage multiplex PCR followed by a second-stage quantitative nested PCR. Raw data were obtained within an hour. All subsequent analyses were performed using normalized expression values.

*Secondary outcome measures*

Secondary outcomes were 28-day, ICU and hospital mortality, ICU free days up to 28 and 90 days, organ support-free days up to 28 days, hospital-free days up to 28 and 90 days, hospital readmissions after discharge up to day-90, new infections post hospital discharge (defined as a new course of antibiotics) up to day-90 and health-related quality of life at day-90 measured with the EQ5D-5L questionnaire. Organ support, ICU and hospital-free days were defined as previously recommended (1).

1. Contentin L, Ehrmann S, Giraudeau B. Heterogeneity in the definition of mechanical ventilation duration and ventilator-free days. Am J Respir Crit Care Med [Internet]. 2014 Apr 15 [cited 2024 Feb 20];189(8):998–1002. Available from: www.atsjournals.org

**Supplementary table S1.** Immune Profiling Panel (IPP) gene transcript composition.

| **Gene** | **Name** | **Function** | **High-risk group, relative expression** |
| --- | --- | --- | --- |
| C3AR1 | Complement C3a receptor 1 | Binding of C3a by the encoded receptor activates chemotaxis, granule enzyme release, superoxide anion production, and bacterial opsonization | ↑ |
| CD177 | CD177 molecule | Plays a role in neutrophil activation and neutrophil transmigration. | ↑ |
| CD3D | CD3d molecule | part of the T-cell receptor/CD3 complex and is involved in T-cell development and signal transduction. | ↓ |
| CD74 | CD74 molecule | Enables MHC class II protein binding activity; cytokine receptor activity; and nitric-oxide synthase binding activity. Acts upstream of or within several processes, including antigen processing and presentation of exogenous peptide antigen via MHC class II; regulation of T cell differentiation; and thymic T cell selection. | ↓ |
| CIITA | Class II major histocompatibility complex transactivator | Acts as a positive regulator of class II major histocompatibility complex gene transcription and is referred to as the "master control factor" for the expression of these genes. | ↓ |
| CTLA4 | Cytotoxic T-lymphocyte associated protein 4 | Member of the immunoglobulin superfamily and encodes a protein which transmits an inhibitory signal to T cells. | ↓ |
| CX3CR1 | C-X3-C motif chemokine receptor 1 | Transmembrane protein and chemokine involved in the adhesion and migration of leukocytes | ↓ |
| IFNγ | Interferon gamma | Critical for innate and adaptive immunity against viral, bacterial and protozoan infections. An important activator of macrophages and inducer of major histocompatibility complex class II molecule expression. | ↓ |
| IL1R2 | Interleukin 1 receptor 2 | Binds interleukin alpha, interleukin beta, and interleukin 1 receptor, type I and acts as a decoy receptor that inhibits the activity of its ligands. | ↑ |
| S100A9 | S100 calcium binding protein A9 | Controls neutrophil and macrophage accumulation, and macrophage cytokine production. Contributes to creating an overall immunosuppressive microenvironment. | ↑ |
| TAP2 | Transporter 2, ATP binding cassette subfamily B member | Involved in antigen presentation. | ↓ |

**Supplementary table S2:** Number of patients recruited by site.

| **Country** | **Hospital** | **n** |
| --- | --- | --- |
| United Kingdom | St Mary’s Hospital London | 75 |
|  | Charing Cross | 60 |
|  | Hammersmith | 34 |
|  | University College | 43 |
| France | Cochin | 24 |
|  | Edouard Herriot | 4 |
|  | Bichat | 12 |
|  | Henri Mondor | 17 |
|  | Georges Pompidou | 1 |
|  | Ambroise Paré | 3 |
|  | Louis Mourier | 8 |
|  | Kremlin-Bicêtre | 13 |
|  | Tenon | 6 |
| Sweden | Karolinska Huddinge | 26 |
|  | Karolinska Solna | 11 |
|  | Sodersjukhuset | 16 |
|  | Danderyd | 4 |

**Supplementary table S3:** Full list of comorbidities by recruiting country. Where the denominator is different from that for the country it is given after the “/”

| **Comorbidity** | **Severity** | **France** | **Sweden** | **United Kingdom** | **Global population** |
| --- | --- | --- | --- | --- | --- |
| n |  | 88 | 57 | 212 | 357 |
| Diabetes mellitus | None or diet controlled | 62 (70.5%) | 41 (71.9%) | 154 (72.6%) | 257 (72.0%) |
|  | Yes | 26 (29.5%) | 16 (28.1%) | 58 (27.4%) | 100 (28.0%) |
|  | End Organ Damage | 6/26 (23.1%) | 7/16 (43.8%) | 16/58 (27.6%) | 29/100 (29.0%) |
| Chronic Lung Disease | Any | 19 (21.6%) | 6 (10.5%) | 24 (11.3%) | 49 (13.7%) |
|  | Severe | 10/19 (52.6%) | 2/6 (33.3%) | 6/24 (25.0%) | 18/49 (36.7%) |
| Cancer | Any | 4 (4.5%) | 2 (3.5%) | 36 (17.0%) | 42 (11.8%) |
|  | Localized | 3/4 (75.0%) | 2/2 (100.0%) | 31/36 (86.1%) | 36/42 (85.7%) |
|  | Metastatic | 1/4 (25.0%) | 0/2 (0.0%) | 5/36 (13.9%) | 6/42 (14.3%) |
| Chronic Renal Failure | Any | 12 (13.6%) | 6 (10.5%) | 18 (8.5%) | 36 (10.1%) |
|  | Moderate to Severe | 10/11 (90.9%) | 5/6 (83.3%) | 9/17 (52.9%) | 24/34 (70.6%) |
|  | Mild | 1/11 (9.1%) | 1/6 (16.7%) | 8/17 (47.1%) | 10/34 (29.4%) |
|  | Dialysis | 3/12 (25.0%) | 2/5 (40.0%) | 4/16 (25.0%) | 9/33 (27.3%) |
| Liver Disease | Any | 7 (8.0%) | 6 (10.5%) | 18 (8.5%) | 31 (8.7%) |
|  | Moderate to Severe | 5/7 (71.4%) | 4/5 (80.0%) | 11/15 (73.3%) | 20/27 (74.1%) |
|  | Mild | 2/7 (28.6%) | 1/5 (20.0%) | 4/15 (26.7%) | 7/27 (25.9%) |
|  | Cirrhosis | 7/7 (100.0%) | 5/6 (83.3%) | 13/18 (72.2%) | 25/31 (80.6%) |
| Peripheral Vascular Disease | Any | 11 (12.5%) | 0 (0.0%) | 14 (6.6%) | 25 (7.0%) |
| Cerebrovascular Accident | Any | 6 (6.8%) | 4 (7.0%) | 13 (6.1%) | 23 (6.4%) |
| Hemiplegia | Any | 1 (1.1%) | 1 (1.8%) | 0 (0.0%) | 2 (0.6%) |
| Myocardial Infarction | Any | 8 (9.1%) | 6 (10.5%) | 6 (2.8%) | 20 (5.6%) |
| Heart Failure | Any | 4 (4.5%) | 7 (12.3%) | 7 (3.3%) | 18 (5.0%) |
|  | Heart Failure Class IV | 0/4 (0.0%) | 1/7 (14.3%) | 2/7 (28.6%) | 3/18 (16.7%) |
| Peptic Ulcer Disease | Any | 3 (3.4%) | 1 (1.8%) | 2 (0.9%) | 6 (1.7%) |
| Connective Tissue Disease | Any | 1 (1.1%) | 0 (0.0%) | 3 (1.4%) | 4 (1.1%) |
| Dementia | Any | 1 (1.1%) | 0 (0.0%) | 2 (0.9%) | 3 (0.8%) |
| Immunocompromised | Any | 0 (0.0%) | 0 (0.0%) | 1 (0.5%) | 1 (0.3%) |
| Leukemia | Any | 0 (0.0%) | 0 (0.0%) | 0 (0.0%) | 0 (0.0%) |
| Lymphoma | Any | 0 (0.0%) | 0 (0.0%) | 0 (0.0%) | 0 (0.0%) |
| AIDS | Any | 0 (0.0%) | 0 (0.0%) | 0 (0.0%) | 0 (0.0%) |

**Supplementary table S4:** Study outcomes by recruiting country. Continuous variables are given as medians and inter-quartile ranges and categorical variables as number and percentage.

|  | **France** | **Sweden** | **United Kingdom** | **Global Population** |
| --- | --- | --- | --- | --- |
| n | 88 | 57 | 212 | 357 |
| **Outcomes** | | | | |
| Day-28 ICU free days | 0 (0-22.0) | 21.0 (9.0-25.0) | 8.0 (0.0-19.0) | 11 (0-22) |
| Day-90 hospital free days | 0 (0-60.2) | 59.0 (25.0-78.0) | 13.0 (0.0-64.0) | 25.0 (0.0-65.0) |
| Day-28 invasive mechanical ventilation free days | 13.5 (0-26.2) | 23.0 (15.0-28.0) | 16.5 (0.0-26.0) | 18 (0-27) |
| Day-28 RRT free days | 23.5 (0.0-28.0) | 28.0 (26.0-28.0) | 28.0 (0.0-28.0) | 28 (0-28) |
| Day-28 vasopressor free days | 18.0 (0-28.0) | 24.0 (15.0-27.0) | 22.0 (0.0-26.0) | 22 (0-27) |
| Day-90 mortality | 38 (43) | 11 (19) | 57 (27) | 106 (30) |
| HAI by day 90 (new course of antibiotics) | 24 (27) | 14 (25) | 60 (28) | 98 (28) |
| HAI by day 90 (adjudicated) | 17 (19) | 11 (19) | 27 (13) | 55 (15) |

**Supplementary table S5:** Source of infection for hospital acquired infections (HAI) defined as either patients started on a new course of antibiotics after ≥48h off antibiotics or as judged as either definite or possible HAIs by the adjudication panel.

| **Infection source** | **HAI by new course of antibiotics** | | | **Adjudicated HAI** | | |
| --- | --- | --- | --- | --- | --- | --- |
|  | Any time | Before day 28 | After day 28 | Any time | Before day 28 | After day 28 |
| **n** | 98 | 81 | 17 | 55 | 44 | 11 |
| **Pneumonia / tracheobronchitis** | 60 (61.2%) | 51 (63.0%) | 9 (52.9%) | 27 (49.1%) | 24 (54.5%) | 3 (27.3%) |
| **Intra-abdominal** | 9 (9.2%) | 8 (9.9%) | 1 (5.9%) | 9 (16.4%) | 8 (18.2%) | 1 (9.1%) |
| **Blood** | 9 (9.2%) | 7 (8.6%) | 2 (11.8%) | 7 (12.7%) | 5 (11.4%) | 2 (18.2%) |
| **Urine** | 11 (11.2%) | 8 (9.9%) | 3 (17.6%) | 7 (12.7%) | 4 (9.1%) | 3 (27.3%) |
| **Soft tissue** | 6 (6.1%) | 5 (6.2%) | 1 (5.9%) | 4 (7.3%) | 3 (6.8%) | 1 (9.1%) |
| **Other** | 3 (3.1%) | 2 (2.5%) | 1 (5.9%) | 1 (1.8%) | 0 (0.0%) | 1 (9.1%) |

**Supplementary table S6:** Outcomes by clinical worsening groups and sampling period. P-values are given from chi squared testing for categorical Wilcox testing for continuous variables, those in bold are significant at p<0.05. Hospital acquired infection (HAI) has been compared with both a competing risk model and where events were censored for HAIs that occurred prior to sampling. (HAI, hospital acquired infection; ICU, intensive care unit; RRT, renal replacement therapy).

|  | **Sample S1 (Day 2-5)** | | | **Sample S2 (Day 6-8)** | | | **Sample S3 (Day 13-15)** | | |
| --- | --- | --- | --- | --- | --- | --- | --- | --- | --- |
|  | **High-risk** | **Low-risk** | **p-value** | **High-risk** | **Low-risk** | **p-value** | **High-risk** | **Low-risk** | **p-value** |
| **n** | 190 | 167 | **-** | 122 | 181 | - | 25 | 144 | - |
| **Death** | | | | | | | | | |
| Day-90 | 66 (35) | 40 (24) | **0.04** | 52 (43) | 36 (20) | **<0.001** | 13 (52) | 34 (24) | **0.007** |
| Day-28 | 49 (26) | 34 (20) | 0.28 | 37 (30) | 29 (16) | **0.005** | 9 (36) | 20 (14) | **0.017** |
| Hospital deaths to day-90 | 63 (33) | 36 (22) | **0.02** | 50 (41) | 31 (17) | **<0.001** | 13 (52) | 30 (21) | **0.002** |
| ICU deaths to day-90 | 42 (22) | 23 (14) | 0.06 | 33 (27) | 20 (11) | **<0.001** | 12 (48) | 13 (9) | **<0.001** |
| **Hospital Acquired Infection** | | | | | | | | | |
| New course of antibiotics | | | | | | | | | |
| Day-28  No event  Death with no HAI  HAI | 103/188 (55)  43/188 (23)  42/188 (22) | 103/161 (64)  27/161 (17)  31/161 (19) | 0.20 | 58/116 (50)  29/116 (25)  29/116 (25) | 115/175 (66)  26/175 (15)  34/175 (19) | **0.02** | 9/17 (53)  5/17 (29)  3/17 (18) | 88/123 (72)  16/123 (13)  19/123 (15) | 0.19 |
| Day-28 HAI censored | 42/188 (22) | 31/161 (19) | 0.57 | 29/116 (25) | 34/175 (19) | 0.33 | 3/17 (18) | 19/123 (15) | 0.73 |
| Adjudicated | | | | | | | | | |
| Day-28  No event  Death with no HAI  HAI | 122/188 (65)  44/188 (23)  22/188 (12) | 119/163 (73)  28/163 (17)  16/163 (10) | 0.25 | 69/116 (60)  30/116 (26)  17/116 (15) | 137/178 (77)  26/178 (15)  15/178 (8) | **0.006** | 11/18 (61)  5/11 (28)  2/11 (11) | 110/136 (81)  16/136 (12)  10/136 (7) | 0.10 |
| Day-28 HAI censored | 22/188 (12) | 16/163 (10) | 0.69 | 17/116 (15) | 15/178 (8) | 0.14 | 2/18 (11) | 10/136 (7) | 0.63 |
| **Treatment and organ support** | | | | | | | | | |
| Invasive ventilation free days to day-28 | 13.5 (0-26) | 21 (0-28) | **0.001** | 7.5 (0-22.8) | 21 (0-27) | **<0.001** | 0 (0-5) | 14.5 (0-23) | **0.004** |
| RRT free days to day-28 | 27.5 (0-28) | 28 (17.5-28) | **0.001** | 24 (0-28) | 28 (23-28) | **<0.001** | 4 (0-28) | 28 (18.8-28) | **0.005** |
| Vasopressor free days to day-28 | 19 (0-26) | 24 (0-27) | **0.003** | 14 (0-24) | 24 (10-27) | **<0.001** | 0 (0-9) | 19.5 (0-26) | **<0.001** |
| Steroid free days to day-28 | 26 (0-28) | 28 (8.5-28) | **0.006** | 23 (0-28) | 28 (21-28) | **<0.001** | 0 (0-28) | 27 (18.8-28) | **0.01** |
| Organ support free days to day -28 | 9.0 (0.0-24.0) | 20.0 (0.0-26.0) | **0.001** | 4.5 (0.0-20.0) | 18.0 (0.0-25.0) | **<0.001** | 0.0 (0.0-4.0) | 11.5 (0.0-20.0) | **0.007** |
| ICU free days to day-28 | 1 (0-19.8) | 16 (0-23) | **<0.001** | 0 (0-15) | 15 (0-22) | **<0.001** | 0 (0-0) | 5.5 (0-16) | **0.001** |
| Hospital free days to day-28 | 0 (0-0) | 0 (0-7.5) | **<0.001** | 0 (0-0) | 0 (0-9) | **<0.001** | 0 (0-0) | 0 (0-0) | 0.11 |
| ICU free days to day-90 | 60.5 (0.0-81.0) | 76.0 (5.0-85.0) | **0.002** | 41.5 (0.0-76.0) | 75.0 (33.0-84.0) | **<0.001** | 0.0 (0.0-61.0) | 63.5 (0.0-77.0) | **0.002** |
| Hospital free days to day-90 | 0.0 (0.0-57.5) | 43.0 (0.0-69.0) | **0.003** | 0.0 (0.0-51.0) | 40.0 (0.0-71.0) | **<0.001** | 0.0 (0.0-12.0) | 6.0 (0.0-48.0) | **0.048** |
| ICU length of stay | 13.0 (6.0-26.5) | 9.0 (4.0-16.5) | **0.001** | 14.0 (8.2-24.5) | 10.0 (6.0-19.0) | **0.003** | 23.0 (15.0-48.0) | 18.0 (11.5-29.2) | 0.09 |
| Hospital length of stay | 30.0 (15.2-62.8) | 23.0 (11.5-49.5) | 0.05 | 30.5 (17.0-58.5) | 26.0 (15.0-64.0) | 0.49 | 34.0 (22.0-68.0) | 49.0 (26.0-90.0) | 0.18 |
| **Post hospital discharge** |  |  |  |  |  |  |  |  |  |
| Readmission to hospital, n | 190  22 (12) | 167  20 (12) | 1.00 | 122  11 (9) | 181  25 (14) | 0.28 | 25  3 (12) | 144  16 (11) | 1.00 |
| Course of antibiotics, n | 85  19 (22) | 90  23 (26) | 0.75 | 46  11 (24) | 101  26 (26) | 0.97 | 7  3 (43) | 65  16 (25) | 0.37 |

**Supplementary table S7:** Clinical worsening model health-related quality of life outcomes (EQ5D-5L) at day-90. P-values are given for Cochran Armitage test for trends.

|  | **Sample S1 (Day 2-5)** | | | **Sample S2 (Day 6-8)** | | | **Sample S3 (Day 13-15)** | | |
| --- | --- | --- | --- | --- | --- | --- | --- | --- | --- |
|  | **High-risk** | **Low-risk** | **p-value** | **High-risk** | **Low-risk** | **p-value** | **High-risk** | **Low-risk** | **p-value** |
| **Anxiety and Depression** | | | | | | | | | |
| n  I am not anxious or depressed  I am slightly anxious or depressed  I am moderately anxious or depressed  I am severely anxious or depressed  I am extremely anxious or depressed | 81  2 (2)  7 (9)  41 (51)  6 (7)  25 (31) | 95  4 (4)  18 (19)  42 (44)  4 (4)  27 (28) | 0.21 | 45  1 (2)  6 (13)  20 (44)  3 (7)  15 (33) | 110  5 (5)  16 (15)  49 (45)  6 (5)  34 (31) | 0.57 | 10  1 (10)  1 (10)  2 (20)  3 (30)  3 (30) | 73  1 (1)  13 (18)  32 (44)  3 (4)  24 (33) | 0.79 |
| **Mobility** | | | | | | | | | |
| n  I have no problems in walking about  I have slight problems in walking about  I have moderate problems in walking about  I have severe problems in walking about  I am unable to walk about | 83  9 (11)  17 (20)  27 (33)  8 (10)  22 (27) | 96  9 (9)  18 (19)  36 (38)  7 (7)  26 (27) | 0.86 | 46  4 (9)  9 (20)  11 (24)  7 (15)  15 (33) | 112  13 (12)  23 (21)  40 (36)  6 (5)  30 (27) | 0.23 | 10  2 (20)  2 (20)  2 (20)  3 (30)  1 (10) | 75  13 (17)  16 (21)  19 (25)  5 (7)  22 (29) | 0.69 |
| **Pain and Discomfort** | | | | | | | | | |
| n  I have no pain or discomfort  I have slight pain or discomfort  I have moderate pain or discomfort  I have severe pain or discomfort  I have extreme pain or discomfort | 82  2 (2)  17 (21)  39 (48)  4 (5)  20 (24) | 96  0 (0)  24 (25)  36 (38)  5 (5)  31 (32) | 0.34 | 46  2 (4)  11 (24)  16 (35)  2 (4)  15 (33) | 111  0 (0)  25 (23)  51 (46)  5 (5)  30 (27) | 0.96 | 10  1 (10)  4 (40)  2 (20)  0 (0)  3 (30) | 75  1 (1)  14 (19)  30 (40)  5 (7)  25 (33) | 0.20 |
| **Self Care** | | | | | | | | | |
| n  I have no problems washing or dressing myself  I have slight problems washing or dressing myself  I have moderate problems washing or dressing myself  I have severe problems washing or dressing myself  I am unable to wash or dress myself | 83  6 (7)  15 (18)  41 (49)  5 (6)  16 (19) | 96  6 (6)  13 (14)  56 (58)  6 (6)  15 (16) | 0.97 | 46  3 (7)  8 (17)  19 (41)  3 (7)  13 (28) | 112  8 (7)  19 (17)  64 (57)  7 (6)  14 (13) | 0.09 | 10  3 (30)  3 (30)  3 (30)  0 (0)  1 (10) | 75  7 (9)  14 (19)  34 (45)  9 (12)  11 (15) | 0.06 |
| **Usual Activities** | | | | | | | | | |
| n  I have no problems doing my usual activities  I have slight problems doing my usual activities  I have moderate problems doing my usual activities  I have severe problems doing my usual activities  I am unable to do my usual activities | 83  18 (22)  18 (22)  22 (27)  9 (11)  16 (19) | 96  21 (22)  12 (13)  24 (25)  7 (7)  32 (33) | 0.13 | 46  8 (17)  13 (28)  8 (17)  5 (11)  12 (26) | 112  29 (26)  13 (12)  28 (25)  11 (10)  31 (28) | 0.95 | 10  4 (40)  1 (10)  1 (10)  3 (30)  1 (10) | 75  18 (24)  13 (17)  14 (19)  8 (11)  22 (29) | 0.40 |

**Supplementary table S8:** Baseline patient characteristics and clinical features at the time of sampling by risk group form the clinical worsening model. Where the number of patients with data differ from the total number of patients n is given in the relevant section. BMI, body mass index; SOFA, sequential organ failure assessment score; APACHE, acute physiology and chronic health evaluation, RRT, renal replacement therapy. *APACHE II scores are those at baseline. ^¥^ chi squared test, ^#^ fisher’s test, ^§^ Wilcox test, ^Ɨ^ students t-test.

|  | **Sample S1 (Day 2-5)** | | | **Sample S2 (Day 6-8)** | | | **Sample S3 (Day 13-15)** | | |
| --- | --- | --- | --- | --- | --- | --- | --- | --- | --- |
|  | **High-risk** | **Low-risk** | **p-value** | **High-risk** | **Low-risk** | **p-value** | **High-risk** | **Low-risk** | **p-value** |
| n | 190 | 167 | - | 122 | 181 | - | 25 | 144 | - |
| Number of males | 134 (71) | 113 (68) | 0.64^¥^ | 80 (66) | 131 (72) | 0.26^¥^ | 15 (60) | 103 (72) | 0.36^¥^ |
| Age (years) | 67 (57-75) | 67 (54-74) | 0.42^§^ | 65.0 (56.2-73.0) | 68.0 (56.0-74.0) | 0.73^§^ | 60.0 (52.0-70.0) | 68.0 (59.0-75.0) | **0.046**^§^ |
| Ethnicity  n  Caucasian  Other  Asian  Black | 133  92 (69)  20 (15)  16 (12)  5 (4) | 135  88 (65)  28 (21)  8 (6)  11 (8) | 0.10^¥^ | 84  55 (66)  14 (17)  12 (14)  3 (4) | 143  96 (67)  28 (20)  9 (6)  10 (7) | 0.19^#^ | 11  7 (64)  3 (27)  1 (9)  0 (0) | 110  76 (69)  21 (19)  6 (6)  7 (6) | 0.75^#^ |
| BMI (kg/m^2^), n | 179  25.9 (22.9-28.5) | 156  27.1 (23.7-31.2) | **0.01**^§^ | 113  26.0 (23.0-28.7) | 170  26.7 (24.0-30.8) | **0.04**^§^ | 23  24.6 (22.8-31.0) | 140  26.5 (24.0-29.2) | 0.52^§^ |
| Admission Type  Medical  Emergency Surgery  Elective Surgery | 134 (71)  44 (23)  12 (6) | 125 (75)  33 (20)  9 (5) | 0.66^¥^ | 85 (70)  30 (25)  7 (6) | 131 (72)  40 (22)  10 (6) | 0.87^¥^ | 22 (88)  2 (8)  1 (4) | 109 (76)  29 (20)  6 (4) | 0.30^#^ |
| Charlson comorbidity score | 3.0 (2.0-4.0) | 3.0 (1.0-5.0) | 0.87^§^ | 3.0 (2.0-4.0) | 3.0 (2.0-5.0) | 0.053^§^ | 3.0 (1.0-4.0) | 3.0 (2.0-4.0) | 0.13^§^ |
| Source of infection  n  Lung  Abdomen  Soft tissue or line  Urine  Neurological  Primary bacteraemia  Other | 189  83 (44)  43 (23)  13 (7)  14 (7)  6 (3)  5 (3)  25 (13) | 167  106 (64)  10 (6)  13 (8)  12 (7)  10 (6)  3 (2)  13 (8) | **<0.001^#^** | 121  57 (47)  32 (26)  8 (7)  6 (5)  4 (3)  3 (3)  11 (9) | 181  101 (56)  20 (11)  13 (7)  15 (8)  11 (6)  5 (3)  15 (8) | **0.04^#^** | 25  10 (40)  9 (36)  1 (4)  2 (8)  1 (4)  0 (0)  2 (8) | 143  76 (53)  18 (13)  16 (11)  6 (4)  8 (6)  5 (4)  14 (10) | 0.13^#^ |
| SOFA score, n | 168  10.0 (7.0-12.0) | 142  7.5 (6.0-9.8) | **<0.001**^§^ | 104  8.0 (6.0-12.0) | 141  8.0 (5.0-10.0) | **0.04**^§^ | 13  9.0 (8.0-11.0) | 94  7.0 (5.0-9.0) | 0.12 ^Ɨ^ |
| APACHE II score*, n | 174  22.0 (18.0-27.0) | 149  21.0 (16.0-26.0) | **0.02**^§^ | 110  22.0 (19.0-26.0) | 162  21.0 (16.0-26.0) | 0.08^§^ | 23  23.0 (19.5-29.5) | 131  23.0 (18.0-26.0) | 0.32^Ɨ^ |
| Lactate (mmol/L), n | 188  1.3 (1.0-1.9) | 162  1.0 (0.8-1.3) | **<0.001**^§^ | 117  1.2 (0.9-1.7) | 161  1.0 (0.8-1.3) | **<0.001**^§^ | 21  1.4 (1.0-2.2) | 109  0.9 (0.7-1.4) | **0.02**^§^ |
| Creatinine (μmol/L), n | 188  110 (65-176) | 165  77 (59-140) | **0.002**^§^ | 121  84 (59-176) | 178  77 (56-126) | 0.31^§^ | 23  73 (47-126) | 137  63 (50-100) | 0.72^§^ |
| Bilirubin (μmol/L), n | 179  13 (7-31) | 156  10 (6-12) | **<0.001**^§^ | 113  14 (7-46) | 163  8 (6-14) | **<0.001**^§^ | 18  18 (9-55) | 125  7 (5-13) | **<0.001**^§^ |
| Platelets (x10^9^/L), n | 184  180.5 (124.0-308.5) | 161  214.0 (129.0-300.0) | 0.52^§^ | 120  217.0 (141.5-356.2) | 177  189.0 (111.0-302.0) | 0.07^§^ | 23  166.0 (121.5-275.0) | 138  201.0 (118.2-337.0) | 0.63^§^ |
| PaO_2_/FiO_2_ (mmHg), n | 187  212 (158-274) | 158  215 (151-285) | 0.77^§^ | 113  175 (128-275) | 152  219 (168-296) | **0.002**^§^ | 19  189 (134-240) | 103  247 (183-315) | **0.048**^§^ |
| Invasive mechanical ventilation, n | 187  146 (78) | 159  101 (64) | **0.004**^¥^ | 120  79 (66) | 181  101 (56) | 0.11^¥^ | 25  15 (60) | 141  59 (42) | 0.14^¥^ |
| RRT, n | 187  43 (23) | 159  16 (10) | **0.002**^¥^ | 120  27 (23) | 181  18 (10) | **0.005**^¥^ | 25  8 (32) | 141  15 (11) | **0.01**^#^ |
| Vasopressors, n | 187  129 (69) | 159  99 (62) | 0.23^¥^ | 120  63 (53) | 181  68 (38) | **0.02**^¥^ | 25  16 (64) | 141  39 (28) | **<0.001**^¥^ |
| Steroids, n | 187  79 (42) | 159  19 (12) | **<0.001**^¥^ | 120  36 (30) | 181  16 (9) | **<0.001**^¥^ | 25  6 (24) | 141  10 (7) | **0.02**^#^ |

|  | **Sample S1 (Day 2-5)** | | | **Sample S2 (Day 6-8)** | | | **Sample S3 (Day 13-15)** | | |
| --- | --- | --- | --- | --- | --- | --- | --- | --- | --- |
|  | **High-risk** | **Low-risk** | **p-value** | **High-risk** | **Low-risk** | **p-value** | **High-risk** | **Low-risk** | **p-value** |
| **n** | 288 | 69 | **-** | 227 | 76 | **-** | 62 | 107 | **-** |
| **Death** | | | | | | | | | |
| Day-90 | 87 (30) | 19 (27) | 0.77 | 77 (34) | 11 (15) | **0.002** | 22 (36) | 25 (23) | 0.13 |
| Day-28 | 67 (23) | 16 (23) | 1.00 | 58 (26) | 8 (11) | **0.01** | 13 (21) | 16 (15) | 0.43 |
| Hospital deaths to day-90 | 82 (29) | 17 (25) | 0.63 | 72 (32) | 9 (12) | **0.001** | 22 (36) | 21 (20) | **0.04** |
| ICU deaths to day-90 | 59 (21) | 6 (9) | **0.04** | 49 (22) | 4 (5) | **0.002** | 16 (26) | 9 (8) | **0.004** |
| **Hospital Acquired Infection** | | | | | | | | | |
| New course of antibiotics | | | | | | | | | |
| Day-90  No event  Death with no HAI  HAI | 141/282 (50)  69/282 (25)  72/282 (26) | 35/67 (52)  14/67 (21)  18/67 (27) | 0.83 | 99/217 (46)  57/217 (26)  61/217 (28) | 47/74 (64)  10/74 (14)  17/74 (23) | **0.02** | 20/49 (41)  11/49 (22)  18/49 (37) | 52/91 (57)  20/91 (22)  19/91 (21) | 0.10 |
| Day-90 HAI censored | 72/282 (26) | 18/67 (27) | 0.95 | 61/217 (28) | 17/74 (23) | 0.48 | 18/49 (37) | 19/91 (21) | 0.07 |
| Day-28  No event  Death with no HAI  HAI | 163/282 (58)  58/282 (21)  61/282 (22) | 43/67 (64)  12/67 (18)  12/67 (18) | 0.63 | 122/217 (56)  47/217 (22)  48/217 (22) | 51/74 (69)  8/74 (11)  15/74 (20) | 0.08 | 31/49 (63)  6/49 (12)  12/49 (25) | 66/91 (73)  15/91 (17)  10/91 (11) | 0.11 |
| Day-28 HAI censored | 61/282 (22) | 12/67 (18) | 0.61 | 48/217 (22) | 15/74 (20) | 0.87 | 12/49 (25) | 10/91 (11) | 0.06 |
| Panel adjudicated | | | | | | | | | |
| Day-90  No event  Death with no HAI  HAI | 172/283 (61)  73/283 (26)  38/283 (13) | 42/68 (62)  15/68 (22)  11/68 (16) | 0.74 | 126/218 (58)  61/218 (28)  31/218 (14) | 56/76 (74)  10/76 (13)  10/76 (13) | **0.02** | 30/52 (58)  12/52 (23)  10/52 (19) | 69/102 (68)  22/102 (22)  11/102 (11) | 0.31 |
| Day-90 HAI censored | 38/283 (13) | 11/68 (16) | 0.70 | 31/218 (14) | 10/76 (13) | 0.97 | 10/52 (19) | 11/102 (11) | 0.23 |
| Day-28  No event  Death with no HAI  HAI | 191/283 (68)  59/283 (21)  33/283 (12) | 50/68 (74)  13/68 (19)  5/68 (7) | 0.52 | 145/218 (67)  48/218 (22)  25/218 (12) | 61/76 (80)  8/76 (11)  7/76 (9) | 0.06 | 38/52 (73)  6/52 (12)  8/52 (15) | 83/102 (81)  15/102 (15)  4/102 (4) | 0.051 |
| Day-28 HAI censored | 33/283 (12) | 5/68 (7) | 0.42 | 25/218 (12) | 7/76 (9) | 0.74 | 8/52 (15) | 4/102 (4) | **0.02** |
| **Treatment and organ support** | | | | | | | | | |
| Invasive ventilation free days to day-28 | 17 (0-26) | 21 (0-28) | 0.09 | 12 (0-24) | 25 (16.8-28) | **<0.001** | 0 (0-16) | 16 (0-25.5) | **<0.001** |
| RRT free days to day-28 | 28 (0-28) | 28 (0-28) | 0.77 | 28 (0-28) | 28 (28-28) | **<0.001** | 24 (0-28) | 28 (20-28) | **0.008** |
| Vasopressor free days to day-28 | 21 (0-26) | 24 (0-27) | 0.13 | 18 (0-25.5) | 25 (20-27) | **<0.001** | 9.5 (0-19.8) | 21 (0-27) | **<0.001** |
| Steroid free days to day-28 | 27 (0-28) | 28 (0-28) | 0.39 | 26 (0-28) | 28 (24-28) | **0.012** | 23.5 (0-28) | 28 (15-28) | **0.02** |
| Organ support free days to day-28 | 14 (0-25) | 20 (0-25) | 0.26 | 9.0 (0.0-23.0) | 23.0 (14.5-26.0) | **<0.001** | 0.0 (0.0-12.5) | 14.0 (0.0-22.0) | **<0.001** |
| ICU free days to day-28 | 8 (0-21) | 16 (0-23) | 0.06 | 2 (0-19) | 19 (12-24) | **<0.001** | 0 (0-8.5) | 8 (0-18.5) | **0.002** |
| Hospital free days to day-28 | 0 (0-2) | 0 (0-10) | 0.08 | 0 (0-0) | 0 (0-13.2) | **<0.001** | 0 (0-0) | 0 (0-0) | **<0.001** |
| ICU free days to day-90 | 68.5 (0.0-83.0) | 75.0 (0.0-84.0) | 0.24 | 61.0 (0.0-79.0) | 80.5 (71.0-85.0) | **<0.001** | 40.0 (0.0-66.2) | 69.0 (13.5-78.0) | **<0.001** |
| Hospital free days to day-90 | 20.0 (0.0-64.0) | 47.0 (0.0-69.0) | 0.23 | 0.0 (0.0-56.0) | 59.5 (0.0-75.0 | **<0.001** | 0.0 (0.0-35.5) | 13.0 (0.0-58.5) | **0.005** |
| ICU length of stay (days) | 11 (5-23) | 8 (4-16) | **0.02** | 13.0 (7.5-26.5) | 9.0 (4.0-16.0) | **<0.001** | 24.0 (15.0-44.5) | 17.0 (9.0-27.0) | **0.005** |
| Hospital length of stay (days) | 27 (14-58) | 21 (13-42) | 0.19 | 30.0 (16.0-68.5) | 21.0 (12.8-46.0) | **0.02** | 49.5 (30.0-90.0) | 43.0 (24.0-78.0) | 0.18 |
| **Post hospital discharge** |  |  |  |  |  |  |  |  |  |
| Readmission to hospital, n | 288  30 (10) | 69  12 (17) | 0.16 | 227  22 (10) | 76  14 (18) | 0.07 | 62  7 (11) | 107  12 (11) | 1.00 |
| Course of antibiotics, n | 136  29 (21) | 39  13 (33) | 0.18 | 98  21 (21) | 49  16 (33) | 0.20 | 21  6 (29) | 51  13 (25) | 0.78 |

**Supplementary table S9:** Outcomes by mHLA-DR model groups and sampling period. P-values are given from chi squared testing for categorical Wilcoxon testing for continuous variables, those in bold are significant at p<0.05. Hospital acquired infection (HAI) has been compared with both a competing risk model and where events were censored for HAIs that occurred prior to sampling. (HAI, hospital acquired infection; ICU, intensive care unit; RRT, renal replacement therapy).

**Supplementary table S10:** mHLA-DR based model health-related quality of life outcomes (EQ5D-5L) at day-90. P-values are given for Cochran Armitage test for trends and those <0.05 are in bold.

|  | **Sample S1 (Day 2-5)** | | | **Sample S2 (Day 6-8)** | | | **Sample S3 (Day 13-15)** | | |
| --- | --- | --- | --- | --- | --- | --- | --- | --- | --- |
|  | **High-risk** | **Low-risk** | **p-value** | **High-risk** | **Low-risk** | **p-value** | **High-risk** | **Low-risk** | **p-value** |
| **Anxiety and Depression** | | | | | | | | | |
| n  I am not anxious or depressed  I am slightly anxious or depressed  I am moderately anxious or depressed  I am severely anxious or depressed  I am extremely anxious or depressed | 140  5 (4)  17 (12)  66 (47)  8 (6)  44 (31) | 36  1 (3)  8 (22)  17 (47)  2 (6)  8 (22) | 0.21 | 109  5 (5)  13 (12)  48 (44)  8 (7)  35 (32) | 46  1 (2)  9 (20)  21 (46)  1 (2)  14 (30) | 0.59 | 29  2 (7)  3 (10)  11 (38)  4 (14)  9 (31) | 54  0 (0)  11 (20)  23 (43)  2 (4)  18 (33) | 0.95 |
| **Mobility** | | | | | | | | | |
| n  I have no problems in walking about  I have slight problems in walking about  I have moderate problems in walking about  I have severe problems in walking about  I am unable to walk about | 142  14 (10)  26 (18)  51 (36)  12 (8)  39 (27) | 37  4 (11)  9 (24)  12 (32)  3 (8)  9 (24) | 0.55 | 111  15 (14)  24 (22)  31 (28)  11 (10)  30 (27) | 47  2 (4)  8 (17)  20 (43)  2 (4)  15 (32) | 0.24 | 30  6 (20)  6 (20)  4 (13)  6 (20)  8 (27) | 55  9 (16)  12 (2)  17 (31)  2 (4)  15 (27) | 0.77 |
| **Pain and Discomfort** | | | | | | | | | |
| n  I have no pain or discomfort  I have slight pain or discomfort  I have moderate pain or discomfort  I have severe pain or discomfort  I have extreme pain or discomfort | 141  2 (1)  31 (22)  63 (45)  7 (5)  38 (27) | 37  0 (0)  10 (27)  12 (32)  2 (5)  13 (35) | 0.49 | 110  2 (2)  27 (25)  45 (41)  7 (6)  29 (26) | 47  0 (0)  9 (19)  22 (47)  0 (0)  16 (34) | 0.37 | 30  1 (3)  11 (37)  11 (37)  1 (3)  6 (20) | 55  1 (2)  7 (13)  21 (38)  4 (7)  22 (40) | **0.01** |
| **Self Care** | | | | | | | | | |
| n  I have no problems washing or dressing myself  I have slight problems washing or dressing myself  I have moderate problems washing or dressing myself  I have severe problems washing or dressing myself  I am unable to wash or dress myself | 142  9 (6)  23 (16)  75 (53)  9 (6)  26 (18) | 37  3 (8)  5 (14)  22 (59)  2 (5)  5 (14) | 0.57 | 111  9 (8)  23 (21)  50 (45)  9 (8)  20 (18) | 47  2 (4)  4 (9)  33 (70)  1 (2)  7 (15) | 0.69 | 30  4 (13)  8 (27)  7 (23)  5 (17)  6 (20) | 55  6 (11)  9 (16)  30 (54)  4 (7)  6 (11) | 0.64 |
| **Usual Activities** | | | | | | | | | |
| n  I have no problems doing my usual activities  I have slight problems doing my usual activities  I have moderate problems doing my usual activities  I have severe problems doing my usual activities  I am unable to do my usual activities | 142  32 (23)  25 (18)  38 (27)  13 (9)  34 (24) | 37  7 (19)  5 (14)  8 (22)  3 (8)  14 (38) | 0.16 | 111  31 (28)  20 (18)  20 (18)  13 (12)  27 (24) | 47  6 (13)  6 (13)  16 (34)  3 (6)  16 (34) | 0.06 | 30  9 (30)  6 (20)  1 (3)  7 (23)  7 (23) | 55  13 (24)  8 (15)  14 (25)  4 (7)  16 (29) | 0.70 |

**Supplementary table S11:** Baseline patient characteristics and clinical features at the time of sampling by risk group form the mHLA-DR model. Where the number of patients with data differ from the total number of patients n is given in the relevant section. BMI, body mass index; SOFA, sequential organ failure assessment score; APACHE, acute physiology and chronic health evaluation; RRT, renal replacement therapy. *APACHE II scores are those at baseline. ^¥^ chi squared test, ^#^ fisher’s test, ^§^ Wilcox test, ^Ɨ^ students t-test.

|  | **Sample S1 (Day 2-5)** | | | **Sample S2 (Day 6-8)** | | | **Sample S3 (Day 13-15)** | | |
| --- | --- | --- | --- | --- | --- | --- | --- | --- | --- |
|  | **High-risk** | **Low-risk** | **p-value** | **High-risk** | **Low-risk** | **p-value** | **High-risk** | **Low-risk** | **p-value** |
| n | 288 | 69 | - | 227 | 76 | - | 62 | 107 | - |
| Number of males | 196 (68) | 51 (74) | 0.42^¥^ | 152 (67) | 59 (78) | 0.11^¥^ | 46 (74) | 72 (67) | 0.44^¥^ |
| Age (years) | 66 (56-74) | 69 (58-74) | 0.42^§^ | 67.0 (57.0-73.5) | 67.5 (53.0-73.0) | 0.92^§^ | 65.0 (59.0-72.0) | 68.0 (58.0-75.0) | 0.52^§^ |
| Ethnicity  n  Caucasian  Other  Asian  Black | 212  142 (67)  37 (18)  20 (9)  13 (6) | 56  38 (70)  11 (20)  4 (7)  3 (5) | 0.97^#^ | 165  106 (64)  29 (18)  19 (12)  11 (7) | 62  45 (73)  13 (21)  2 (3)  2 (3) | 0.17^#^ | 39  28 (72)  7 (18)  2 (5)  2 (5) | 82  55 (67)  17 (21)  5 (6)  5 (6) | 0.98^#^ |
| BMI (kg/m^2^), n | 272  26.3 (23.1-29.4) | 63  25.8 (23.6-31.7) | 0.60^§^ | 213  26.5 (23.3-29.4) | 70  26.1 (23.8-30.8) | 0.45^§^ | 59  25.5 (23.7-29.3) | 104  26.8 (23.8-29.1) | 0.40^§^ |
| Admission Type  Medical  Emergency Surgery  Elective Surgery | 204 (71)  67 (23)  17 (6) | 55 (80)  10 (15)  4 (6) | 0.30^#^ | 166 (73)  48 (21)  13 (6) | 50 (66)  22 (29)  4 (5) | 0.38^#^ | 48 (77)  11 (18)  3 (5) | 83 (78)  20 (19)  4 (4) | 0.95^#^ |
| Charlson comorbidity score | 3.0 (1.0-4.0) | 4.0 (1.0-5.0) | 0.14^§^ | 3.0 (2.0-4.0) | 3.0 (1.0-5.0) | 0.51^§^ | 3.0 (2.0-4.0) | 3.0 (2.0-4.0) | 0.73^§^ |
| Source of infection  n  Lung  Abdomen  Soft tissue or line  Urine  Neurological  Primary bacteremia  Other | 287  149 (52)  52 (18)  18 (6)  18 (6)  11 (4)  6 (2)  33 (12) | 69  40 (58)  1 (1)  8 (12)  8 (12)  5 (7)  2 (3)  5 (7) | **<0.001**^#^ | 226  117 (52)  46 (20)  14 (6)  14 (6)  13 (6)  4 (2)  18 (8) | 76  42 (55)  6 (8)  7 (9)  7 (9)  2 (3)  4 (5)  8 (11) | 0.07^#^ | 62  29 (47)  19 (31)  4 (7)  3 (5)  1 (2)  0 (0)  6 (10) | 106  57 (54)  8 (8)  13 (12)  5 (5)  8 (8)  5 (5)  10 (9) | **0.003**^#^ |
| **Disease severity, clinical parameters and organ support** | | | | | | | | | |
| SOFA score, n | 252  9.0 (7.0-11.0) | 58  7.5 (5.0-10.0) | **0.014**^§^ | 191  8.0 (5.5-11.0) | 54  8.0 (5.0-10.0) | 0.17^§^ | 40  8.5 (6.8-10.0) | 67  7.0 (5.0-9.0) | **0.03**^Ɨ^ |
| APACHE II score*, n | 263  22.0 (17.0-26.0) | 60  20.0 (16.0-26.0) | 0.19^§^ | 207  22.0 (18.0-26.0) | 65  20.0 (16.0-26.0) | 0.09^§^ | 56  23.5 (19.0-28.0) | 98  22.0 (18.0-25.8) | 0.31^Ɨ^ |
| Lactate (mmol/L), n | 284  1.2 (0.9-1.7) | 66  1.0 (0.8-1.3) | **<0.001**^§^ | 215  1.2 (0.9-1.6) | 63  0.8 (0.7-1.1) | **<0.001**^§^ | 53  1.2 (0.8-1.9) | 77  0.9 (0.7-1.3) | **0.017**^§^ |
| Creatinine (μmol/L), n | 284  92 (62-160) | 69  80 (61-148) | 0.39^§^ | 225  80 (57-152) | 74  77 (58-122) | 0.73^§^ | 59  70 (48-120) | 101  63 (50-94) | 0.60^§^ |
| Bilirubin (μmol/L), n | 270  11 (7-26) | 65  8 (6-11) | **<0.001**^§^ | 210  10 (6-24) | 66  7 (6-11) | **<0.001**^§^ | 52  10 (7-25) | 91  6 (5-11) | **0.001**^§^ |
| Platelets (x10^9^/L), n | 278  194 (129-320) | 67  214 (127.5-288) | 0.97^§^ | 222  207.5 (127.2-329.5) | 75  174.0(114.0-308.5) | 0.47^§^ | 60  203.0 (122.0-302.8) | 101  196.0 (111.0-334.0) | 0.67^§^ |
| PaO_2_/FiO_2_ (mmHg), n | 282  214 (153-273) | 63  205 (155-324) | 0.42^§^ | 208  200 (138-285) | 57  223 (167-300) | 0.15^§^ | 49  197 (153-272) | 73  260 (200-322) | **0.001**^§^ |
| Invasive Mechanical ventilation, n | 279  208 (75) | 67  39 (58) | **0.01**^¥^ | 225  144 (64) | 76  36 (47) | **0.02**^¥^ | 61  33 (54) | 105  41 (39) | 0.09^¥^ |
| RRT | 279  53 (19) | 67  6 (9) | 0.07^¥^ | 225  41 (18) | 76  4 (5) | **0.01**^¥^ | 61  13 (21) | 105  10 (10) | 0.06^¥^ |
| Vasopressors | 279  188 (67) | 67  40 (60) | 0.29^¥^ | 225  104 (46) | 76  27 (36) | 0.14^¥^ | 61  33 (54) | 105  22 (21) | **<0.001**^¥^ |
| Steroids | 279  92 (33) | 67  6 (9) | **<0.001**^¥^ | 225  44 (20) | 76  8 (11) | 0.10^¥^ | 61  10 (16) | 105  6 (6) | **0.048**^¥^ |

**Supplementary table S12:** Outcome measures by trajectory between risk groups over the first two sampling time points by clinical worsening and the mHLA-DR models. ICU, intensive care unit; HAI, hospital acquired infection; RRT, renal replacement therapy. Pairs of letters represent transition between high (H) and low (L) risk groups, for example HH represents a patient who says in the high-risk group.

|  | **Clinical-worsening model** | | | | | **mHLA-DR model** | | | | |
| --- | --- | --- | --- | --- | --- | --- | --- | --- | --- | --- |
|  | **HH** | **LH** | **HL** | **LL** | **p-value** | **HH** | **LH** | **HL** | **LL** | **p-value** |
| n | 108 | 14 | 60 | 121 | - | 212 | 15 | 38 | 38 | - |
| Day-28 mortality | 32 (30) | 5 (36) | 11 (18) | 18 (15) | **0.02** | 54 (26) | 4 (27) | 3 (8) | 5 (13) | **0.04** |
| Day-90 mortality | 47 (44) | 5 (36) | 12 (20) | 24 (20) | **<0.001** | 73 (34) | 4 (27) | 3 (8) | 8 (21) | **0.003** |
| ICU free days to day-28 | 0.0 (0.0-15.2) | 2.5 (0.0-12.0) | 10.0 (0.0-22.0) | 17.0 (0.0-23.0) | **<0.001** | 1.0 (0.0-18.2) | 11.0 (0.0-19.5) | 19.5 (13.2-24.0) | 18.0 (9.5-23.0) | **<0.001** |
| ICU free days to day-90 | 38.0 (0.0-76.2) | 64.0 (0.0-74.0) | 72.0 (39.8-84.0) | 78.0 (27.0-84.0) | **<0.001** | 60.5 (0.0-79.0) | 73.0 (22.5-81.5) | 81.5 (75.2-86.0) | 78.0 (11.8-84.8) | **<0.001** |
| Hospital free days to day-28 | 0.0 (0.0-0.0) | 0.0 (0.0-0.0) | 0.0 (0.0-9.2) | 0.0 (0.0-7.0) | **<0.001** | 0.0 (0.0-0.0) | 0.0 (0.0-0.5) | 6.0 (0.0-16.0) | 0.0 (0.0-12.8) | **<0.001** |
| Hospital free days to day-90 | 0.0 (0.0-48.0) | 22.5 (0.0-54.0) | 37.0 (0.0-71.2) | 43.0 (0.0-69.0) | **<0.001** | 0.0 (0.0-55.2) | 12.0 (0.0-62.0) | 68.0 (19.0-78.0) | 53.5 (0.0-72.8) | **<0.001** |
| Invasive mechanical ventilation free days to day-28 | 7.0 (0.0-23.0) | 8.5 (0.0-18.0) | 17.5 (0.0-26.0) | 22.0 (4.0-28.0) | **<0.001** | 10.0 (0.0-24.2) | 17.0 (0.0-22.5) | 25.0 (20.0-28.0) | 23.0 (15.5-28.0) | **<0.001** |
| RRT free days to day-28 | 23.5 (0.0-28.0) | 28.0 (0.0-28.0) | 28.0 (15.0-28.0) | 28.0 (25.0-28.0) | **<0.001** | 28.0 (0.0-28.0) | 28.0 (0.0-28.0) | 28.0 (28.0-28.0) | 28.0 (23.5-28.0) | **0.002** |
| Vasopressor free days to day-28 | 13.0 (0.0-24.0) | 16.0 (0.0-24.8) | 24.0 (5.2-26.2) | 24.0 (13.0-27.0) | **<0.001** | 17.5 (0.0-25.0) | 23.0 (8.5-27.0) | 26.0 (24.0-27.0) | 24.5 (16.0-28.0) | **<0.001** |
| Steroid free days to day-28 | 23.0 (0.0-28.0) | 24.5 (0.0-28.0) | 27.5 (23.2-28.0) | 28.0 (21.0-28.0) | **<0.001** | 26.0 (0.0-28.0) | 28.0 (12.5-28.0) | 28.0 (25.0-28.0) | 27.5 (19.5-28.0) | **0.047** |
| Organ support free days to day-28 | 3.0 (0.0-20.2) | 8.0 (0.0-17.0) | 13.0 (0.0-24.2) | 20.0 (0.0-25.0) | **<0.001** | 7.5 (0.0-23.0) | 17.0 (0.0-22.0) | 24.0 (16.0-26.0) | 21.0 (0.0-25.0) | **<0.001** |
| ICU length of stay | 14.0 (8.8-27.0) | 16.5 (8.2-19.8) | 13.5 (6.0-28.0) | 9.0 (5.0-16.0) | **0.008** | 13.0 (8.0-27.0) | 12.0 (7.0-17.5) | 8.5 (4.0-13.2) | 9.0 (5.0-16.0) | **<0.001** |
| Hospital length of stay | 31.5 (17.0-63.5) | 26.5 (19.2-51.0) | 27.0 (15.0-67.5) | 26.0 (14.0-58.0) | 0.73 | 31.5 (16.0-68.2) | 27 (16.5-60.5) | 19.5 (12.0-46.8) | 22.0 (14.2-44.2) | 0.15 |
| HAI adjudicated day-28  No event  Dead no HAI  HAI | 62/106 (59)  27/106 (26)  17/106 (16) | 7/14 (50)  3/14 (21)  4/14 (29) | 45/60 (75)  11/60 (18)  4/60 (7) | 92/118 (78)  15/118 (13)  11/118 (9) | **0.02** | 134/208 (64)  46/208 (22)  28/208 (14) | 11/14 (79)  2/14 (14)  1/14 (7) | 31/38 (82)  3/38 (8)  4/38 (11) | 30/38 (79)  5/38 (13)  3/38 (8) | 0.29 |
| HAI adjudicated day-90  No event  Dead no HAI  HAI | 49/106 (46)  37/106 (35)  20/106 (19) | 7/14 (50)  3/14 (21)  4/14 (29) | 43/60 (72)  12/60 (20)  5/60 (8) | 83/118 (70)  19/118 (16)  16/118 (14) | **0.002** | 117/208 (56)  59/208 (28)  32/208 (15) | 9/14 (64)  2/14 (14)  3/14 (21) | 31/38 (82)  3/38 (8)  4/38 (11) | 25/38 (66)  7/38 (18)  6/38 (16) | 0.06 |
| HAI new course of antibiotics day-28  No event  Dead no HAI  HAI | 51/106 (48)  26/106 (25)  29/106 (27) | 7/14 (50)  3/14 (21)  4/14 (29) | 38/60 (63)  11/60 (18)  11/60 (18) | 77/116 (66)  15/116 (13)  24/116 (21) | 0.13 | 113/208 (54)  45/208 (22)  50/208 (24) | 9/14 (64)  2/14 (14)  3/14 (21) | 26/37 (70)  3/37 (8)  8/37 (22) | 25/37 (68)  5/37 (14)  7/37 (19) | 0.41 |
| HAI new course of antibiotics day-90  No event  Dead no HAI  HAI | 35/106 (33)  35/106 (33)  36/106 (34) | 7/14 (50)  3/14 (21)  4/14 (29) | 34/60 (57)  11/60 (18)  15/60 (25) | 70/116 (60)  18/116 (16)  28/116 (24) | **0.002** | 93/208 (45)  55/208 (26)  60/208 (29) | 6/14 (43)  2/14 (14)  6/14 (43) | 26/37 (70)  3/37 (8)  8/37 (22) | 21/37 (57)  7/37 (19)  9/37 (24) | 0.06 |

**Supplementary Figure S1:** Diagram showing the timing of patient sampling in relationship to ICU admission. S1, S2, S3 represent the three sampling timepoints.

**
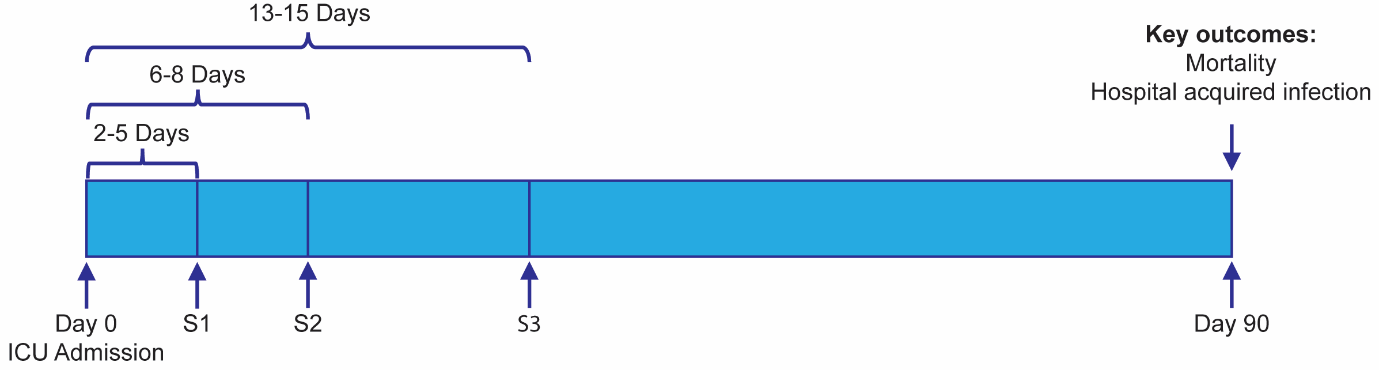
**

**Supplementary Figure S2:** Box and whisker plots comparing the normalized gene expression between the ‘high-risk’ (red) and ‘low-risk’ (blue) groups across the three sampling timepoints, day 2-5 (S1), day 6-8 (S2) and day 13-15 (S3). A) Clinical worsening model and B) mHLA-DR model

**
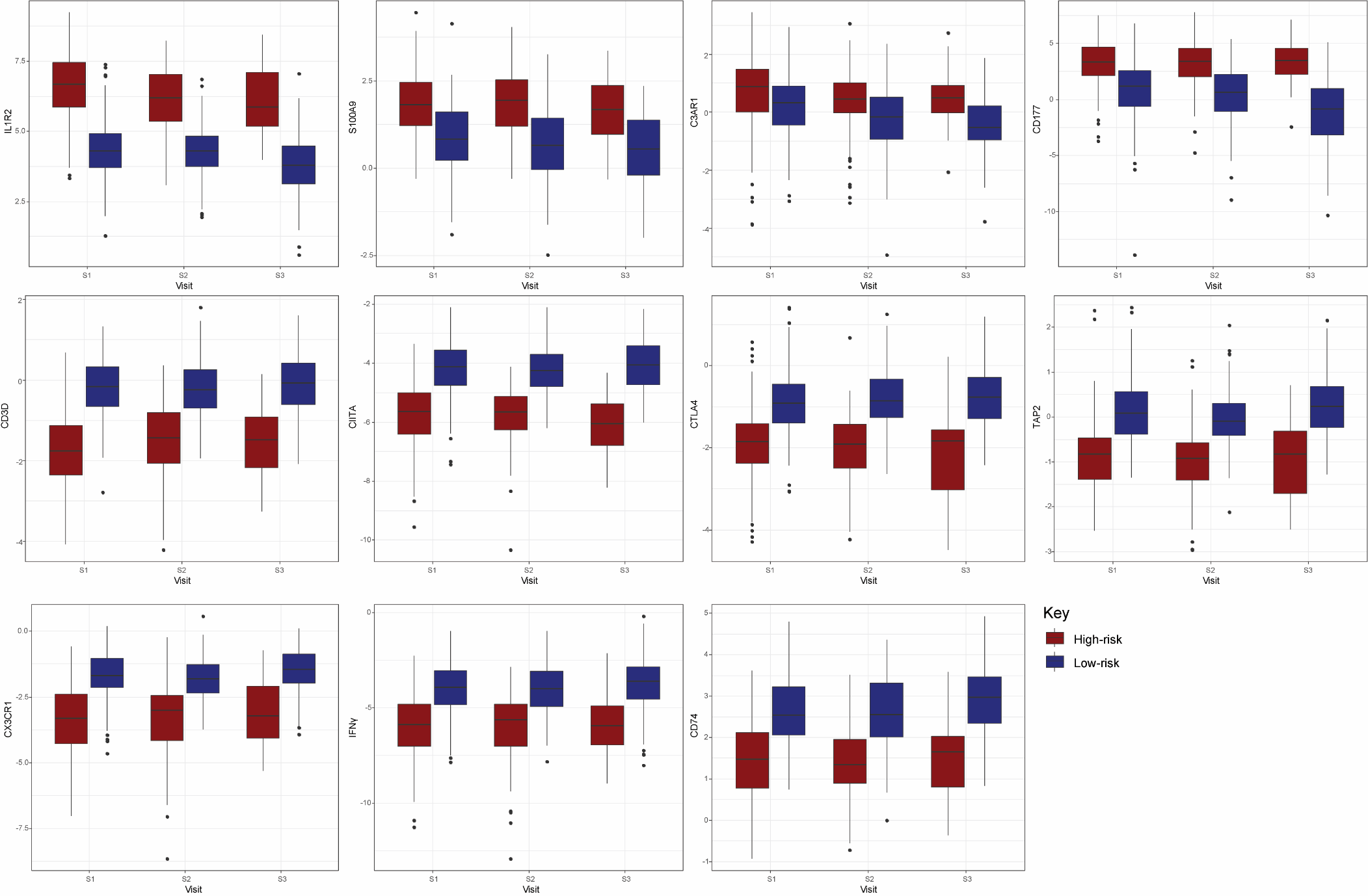
A.**

**B.
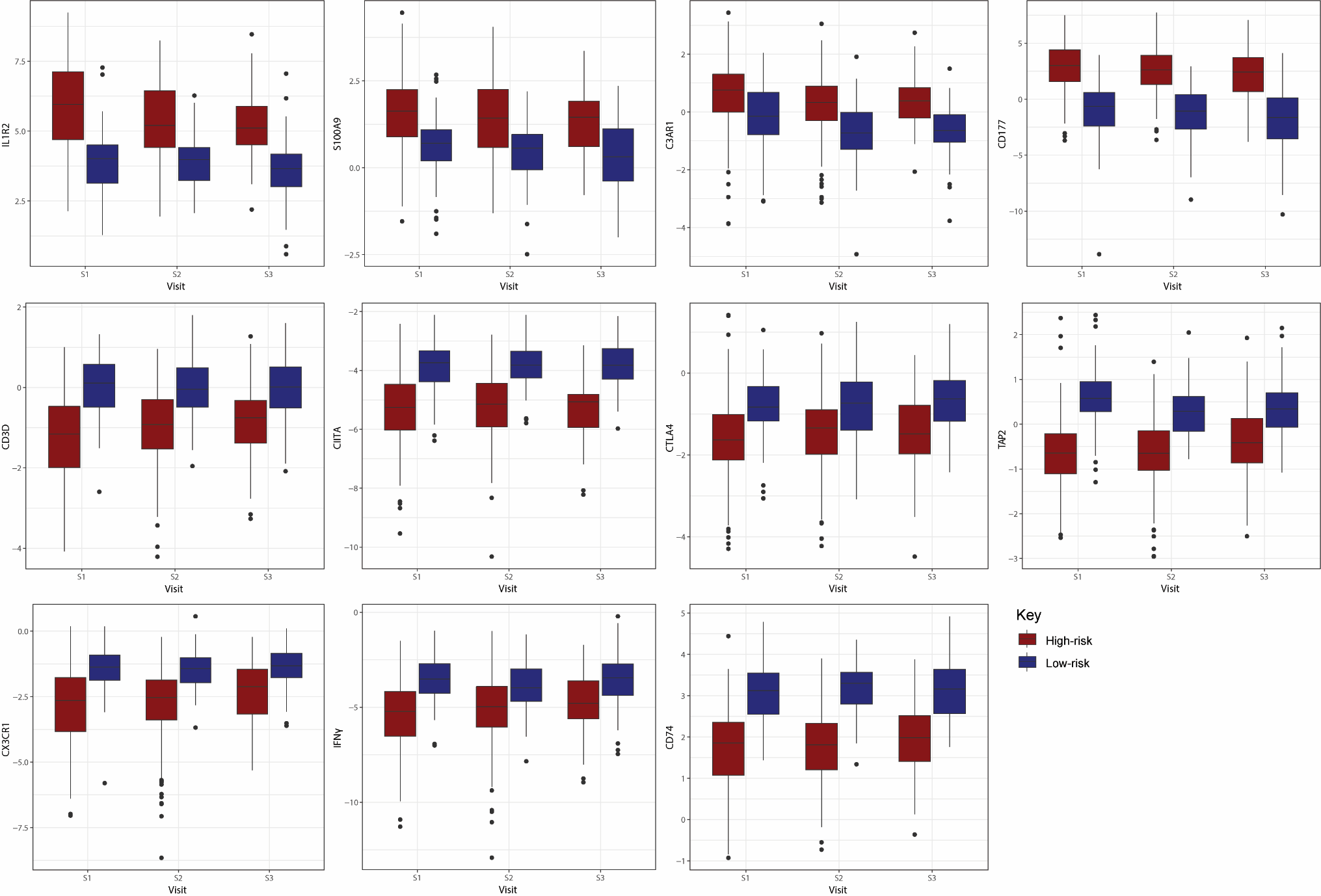
**

**Supplementary Figure S3:** A) Proportion of patients in the high-risk group by sampling time and IPP model (blue – clinical worsening model, red – mHLA-DR model). Alluvial plots showing transition between risk groups over the first two sampling time points (S1, S2, S3) and status at day-90 for B) the clinical worsening model and C) the mHLA-DR model. Colors represent risk group and outcome: low-risk – blue, high-risk – red, discharged – light green, no sample – orange, alive – green, still in hospital – black, still in intensive care (ICU) – purple, dead – grey.

**
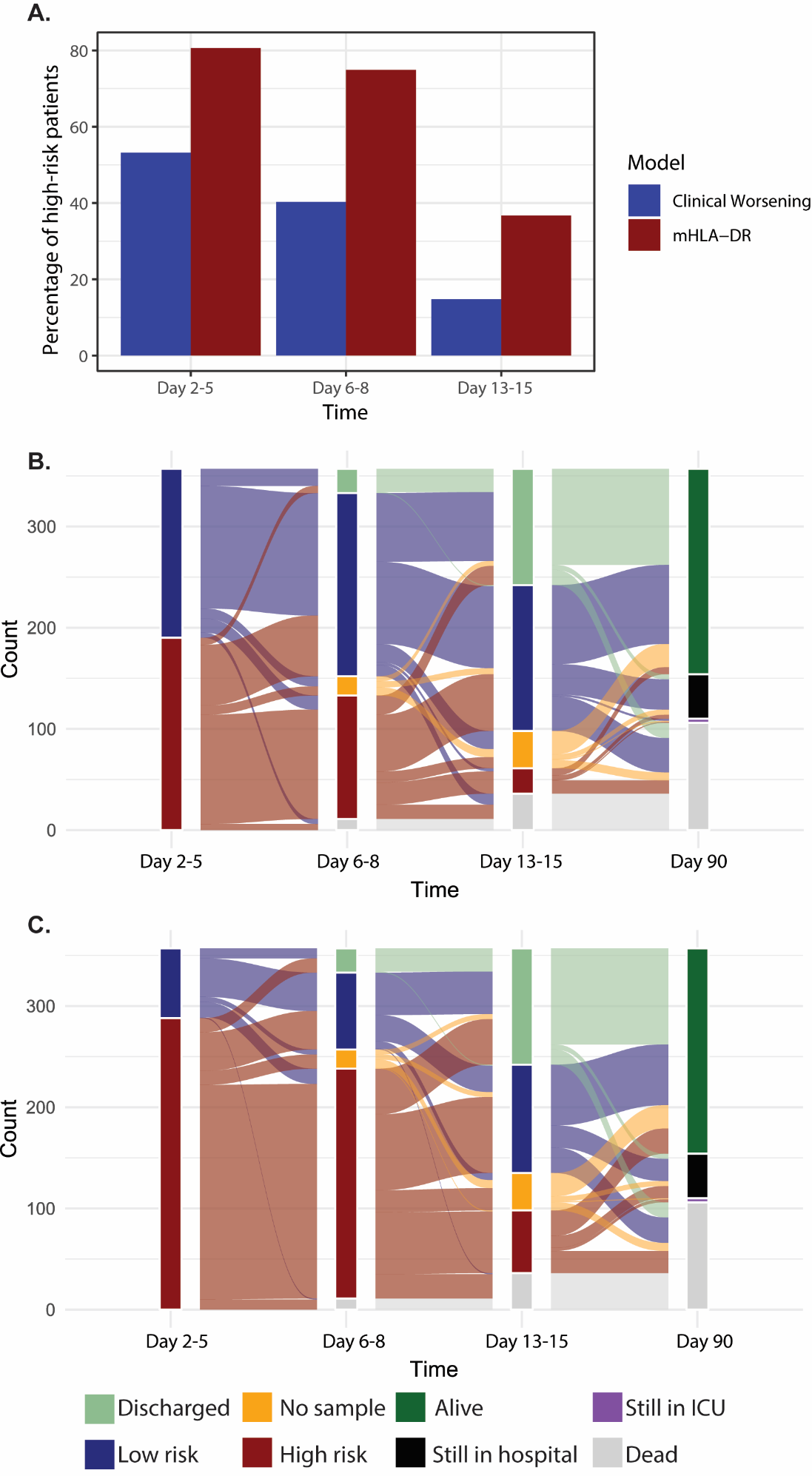
**

**Supplementary Figure S4:** Important baseline characteristics that were significant between the high and low-risk clinical worsening gene-expression groups.


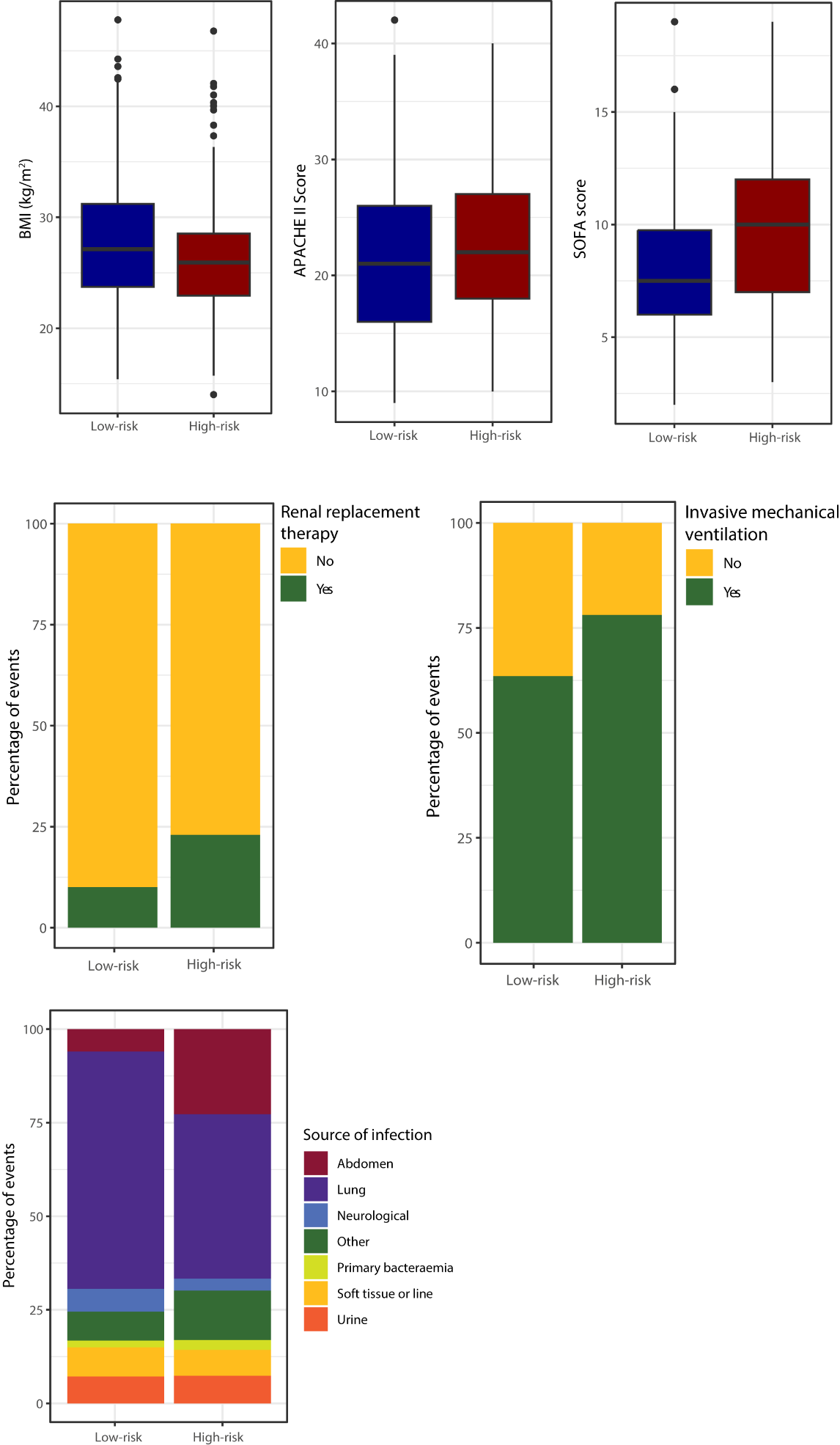


**Supplementary Figure S5:** Receiver operating characteristic curves and area under the receiver operating characteristic cure (AUROC) for the predictive capacity of clinical parameters recorded at the same time as IPP blood sampling (day 2-5, day 6-8 and day 13-15) to identify the IPP high-risk group for both the clinical worsening and mHLA-DR model. (Sequential Organ Failure Assessment score (SOFA)). The AUROC is given for all variable to predict the high-risk group except for PaO_2_/FiO_2_ ratio where the relationship with IPP group is inverted.


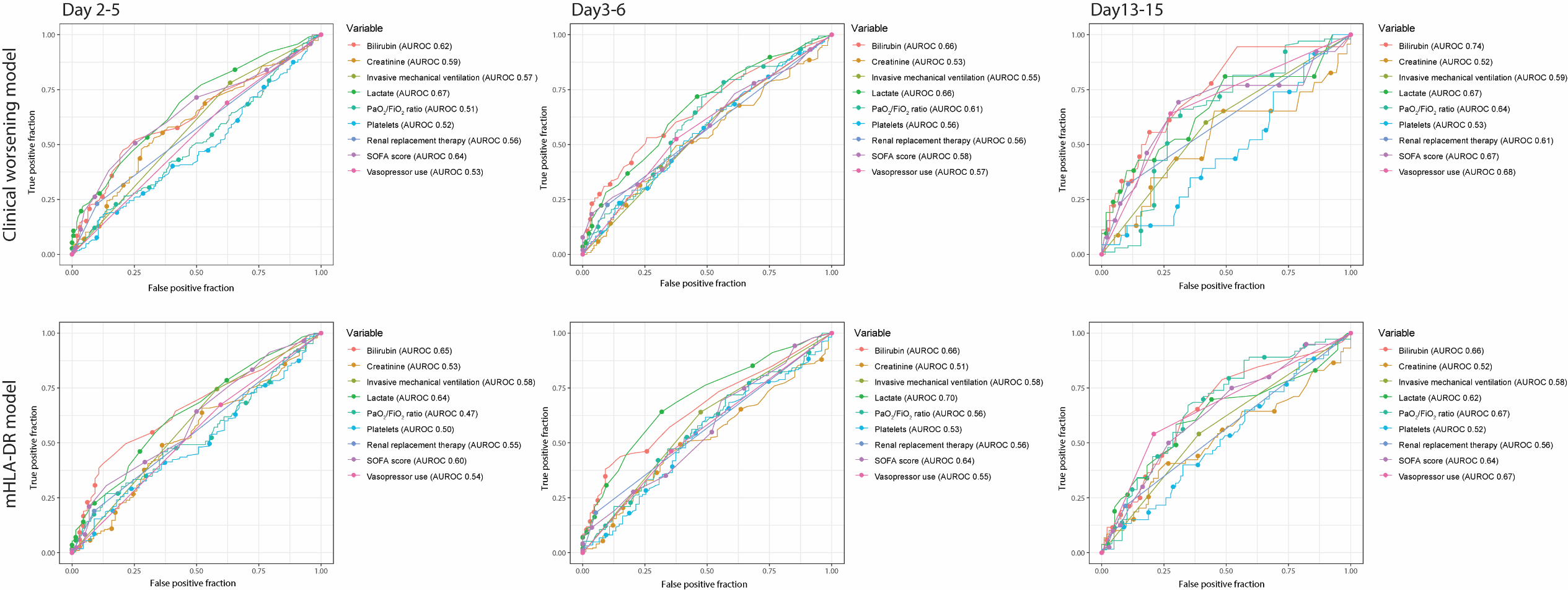


**Supplementary Figure S6:** Bar plots showing A) the proportion of patients with each trajectory between ‘high-risk’ (H) and ‘low-risk’ (L) groups across all sampling time points for both the clinical worsening (blue) and mHLA-DR (red) models and B) the proportion with each trajectory that had died by day-90.


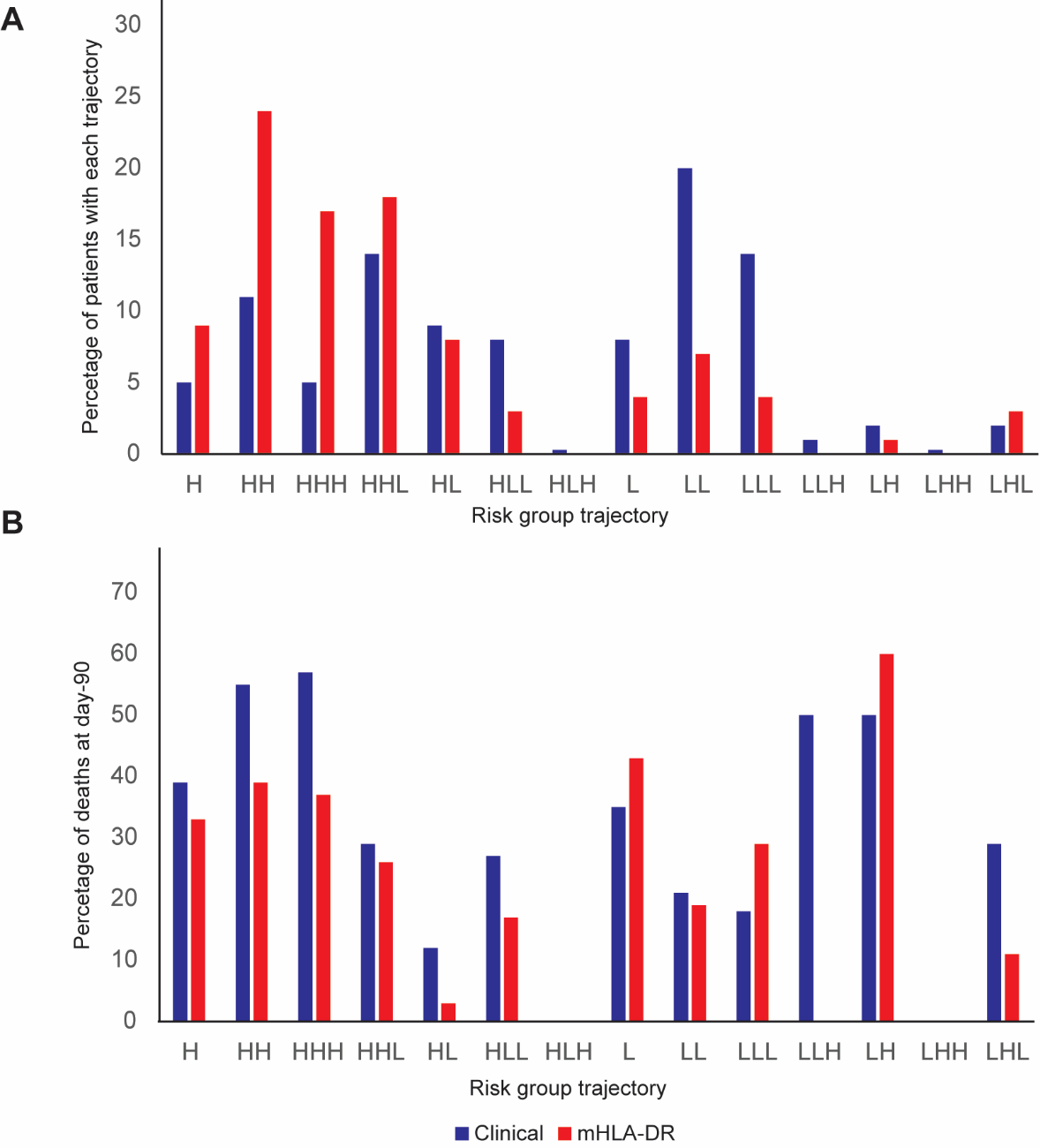

Supplement: Supplementary file 1 — Additional file 1 [file 13054_2025_5319_MOESM1_ESM.docx]
